# Sensory feedback synchronizes motor and sensory neuronal networks in the neonatal rat spinal cord

**DOI:** 10.1038/ncomms13060

**Published:** 2016-10-07

**Authors:** Ana R. Inácio, Azat Nasretdinov, Julia Lebedeva, Roustem Khazipov

**Affiliations:** 1 INMED, INSERM UMR 901, Marseille 13009, France; 2 Aix Marseille Université, Faculté des Sciences, Marseille F-13000, France; 3 Laboratory of Neurobiology, Kazan Federal University, 42008 Kazan, Russia

## Abstract

Early stages of sensorimotor system development in mammals are characterized by the occurrence of spontaneous movements. Whether and how these movements support correlated activity in developing sensorimotor spinal cord circuits remains unknown. Here we show highly correlated activity in sensory and motor zones in the spinal cord of neonatal rats *in vivo*. Both during twitches and complex movements, movement-generating bursts in motor zones are followed by bursts in sensory zones. Deafferentation does not affect activity in motor zones and movements, but profoundly suppresses activity bursts in sensory laminae and results in sensorimotor uncoupling, implying a primary role of sensory feedback in sensorimotor synchronization. This is further supported by largely dissociated activity in sensory and motor zones observed in the isolated spinal cord *in vitro*. Thus, sensory feedback resulting from spontaneous movements is instrumental for coordination of activity in developing sensorimotor spinal cord circuits.

## Abstract (web summary)

Spontaneous movements are important for mammalian development but how network activity underlies the generation of these actions remains unclear. Here the authors show that both spontaneous twitches and complex movements enable correlated activity in motor and sensory networks of the rat spinal cord *in vivo*, and that sensory feedback is instrumental in this synchronization.

## Graphical abstract

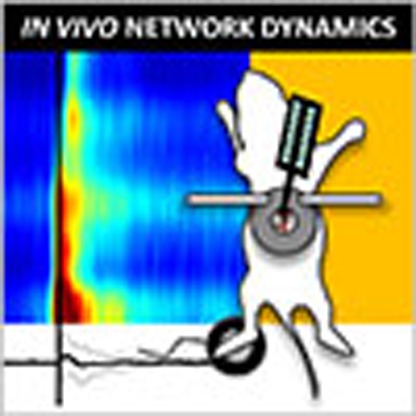

Spontaneous movements are a hallmark of early stages of mammalian development^1^,^2^,^3^. These largely uncoordinated early movement patterns are generated by motor commands from the spinal cord^4^, with a developmental control from the brainstem^5^, notably red nucleus^6^, and persist after decerebration^5^ or deafferentation^2^,^7^. Growing evidence indicates that spontaneous movements are implicated in a number of activity-dependent developmental processes including formation of connections between motoneurons and muscles^8^, development of sensorimotor spinal cord circuits and reflexes^9^,^10^, and formation of thalamocortical body maps^11^,^12^,^13^,^14^,^15^,^16^,^17^,^18^. In rodent embryos, generation of early motor activities in the spinal cord was shown to involve transient circuits, electrical synapses and excitatory actions of GABA^19^,^20^. Similar early movement patterns implicated in a variety of developmental processes also have been described in non-mammalian species, notably chick embryos^19^,^21^ and crustaceans (for review see Marder and Rehm^22^). Yet, the network activity patterns that underlie the generation of motor commands and support sensorimotor coordination in spinal cord circuits during spontaneous motor behaviours in rodents during the postnatal period, which corresponds to the second half of gestation in humans^23^, remain largely unknown.

Current knowledge on the function of the neonatal rodent spinal cord in relation to early movements is mainly based on behavioural and network modelling approaches^9^,^24^,^25^,^26^. These studies suggest important roles of sensory feedback resulting from spontaneous movements in tuning sensorimotor connectivity. Through a series of elegant studies, Schouenborg and colleagues^9^,^26^,^27^ have developed a model of motor-directed somatosensory imprinting to explain the functional adaption of the nociceptive withdrawal reflex, which occurs through the postnatal period in rats. The model applies an anti-Hebbian rule to self-organize sensory-motor connections within nociceptive withdrawal reflex modules in the spinal cord (with postsynaptic activity in movement-generating neurons preceding sensory feedback). Consistently, aberrant sensory input artificially introduced during spontaneous twitches increased the nociceptive withdrawal reflex error rate^9^. These behavioural observations and model predictions of spinal cord circuit function also are supported by results obtained for higher sensorimotor stations (relay thalamus and sensorimotor cortex), where sensory feedback from spontaneous twitches and reflexive responses was shown to trigger internally generated thalamocortical spindle-burst and early gamma oscillations (EGOs)^11^,^12^,^13^,^14^,^17^,^18^,^28^ (see also refs 15, 16). Spontaneous movements also were shown to be efficient triggers of hippocampal activity in neonatal animals^29^. Taken together, the existing evidence, based on behavioural observations and model studies at the level of spinal cord and recordings of neuronal activity at higher brain centres, suggests that sensory feedback is central to the roles fulfilled by spontaneous twitches in coordinating sensorimotor spinal cord networks. However, spinal cord activity in the context of spontaneous movements has never been previously addressed directly. Moreover, several observations question the ubiquity of the sensory feedback hypothesis in relation to spontaneous movements. First, recordings of neuronal activity in cortex of neonatal rats revealed that different types of spontaneous movements evoke cardinally different cortical responses: while twitches and startles occurring during sleep were followed by excitation of neurons in primary motor cortex and hippocampus (in agreement with the sensory feedback hypothesis), complex, long-lasting movements occurring during awake states were not associated with cortical activation^30^. It has been suggested that these two types of motor events are differently processed in cortex, the twitches being processed as unexpected events, whereas expected sensory input during self-generated complex movements is gated via corollary discharges^31^. Gating of sensory feedback could occur at the level of spinal cord, for example via local GABAergic interneuron mediated inhibitory corollary discharge on dorsal sensory neurons and primary afferents^32^, but this remains hypothetical. Even more complexity is added by observations made in the *in vitro* spinal cord preparation, which displays spontaneous motor bursts^33^, and in which other forms of communication between motor and sensory neurons have been suggested, including excitatory efferent copy, mediated either by interneurons activating primary afferents via depolarizing GABA actions or through ephaptic interactions^34^,^35^. However, it remains unknown whether sensorimotor network activity and the local interactions described in the *in vitro* spinal cord preparation correspond to the spinal cord network dynamics in behaving animals.

In this study, we addressed the spatiotemporal dynamics of spinal cord circuitry in relation to neonatal spontaneous movements, *in vivo*, through simultaneous recordings of translaminar spinal cord network activity and spontaneous movements in spine-restrained behaving neonatal rats. We also compared, using a similar approach, the *in vivo* activity with the activity patterns expressed in the isolated spinal cord *in vitro*. Our main finding is that both spontaneous twitches and complex movements enable correlated activity in motor and sensory networks of the spinal cord *in vivo*, and that sensory feedback is instrumental in this synchronization. We also find that although the isolated spinal cord *in vitro* displays spontaneous bursting activities in sensory and motor zones, their spatiotemporal dynamics poorly matches that of the spinal cord *in vivo*, even after deafferentation. These findings provide, for the first time, a description of sensorimotor spinal cord dynamics in relation to neonatal twitches and complex movements, indicate supraspinal mechanisms of inhibition of cortical activation during complex movements, and also show that sensory feedback from twitches indeed creates conditions for a reversed-type of Hebbian learning, with the activity in movement-generating networks preceding the afferent input, which supports the Schouenborg’s model of motor-directed somatosensory imprinting.

## Results

### Functional probing of the neonatal rat spinal cord *in vivo*

**Figure 1:**
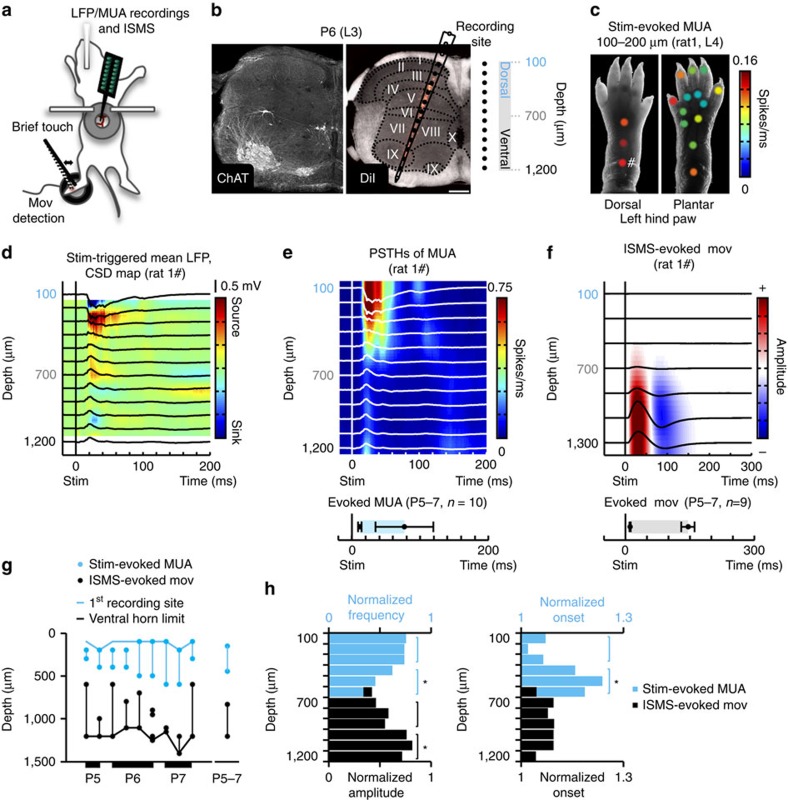
*In vivo* functional mapping of sensory-motor spinal cord zones. (**a**) Schematic illustration of the experimental setup. We recorded spinal cord, translaminar network events, either evoked by mechanical stimulation or spontaneous (and in association with motor behaviour), following which we performed ISMS. (**b**) Silicone-based electrode array track visualized on a transverse spinal cord section (P6 rat). Left: ChAT^+^ cells—motoneurons (Alexa633, confocal image). Expression profiles of ChAT^+^ and NeuN^+^ cells were used to estimate borders of different spinal cord laminae (Supplementary Fig. 1). Right: Overlaid dark-field image and epi-fluorescence image (DiI, red) of the same slice and to-scale scheme of the electrode array (100 μm between recording sites), denoting the approximate anatomical location each recording site (dashed lines—Rexed laminae borders). Scale bar: 200 μm. (**c**) Mechanical stimulus (stim)-evoked MUA frequency represented as colour-coded dot overlaid on the corresponding stimulation point in a aged-matched hindlimb photograph (response map, single, anesthetized animal). Note that all firing frequencies correspond to the two recording sites showing the shortest response onsets, which were consistent across all stimulation points (depths of 100 and 200 μm). The response characterized by the highest MUA frequency (marked by #) was considered topographic and is described in more detail in **d**–**e**. (**d**) Stim-triggered mean LFP traces and CSD map (*n*=100 stimuli, single, non-anesthetized animal). (**e**) Corresponding normalized translaminar peri-stim time-histograms (PSTHs) of MUA. Note the duration of response across non-anesthetized animals (mean±s.d., *n*=10). A comparative description of sensory-evoked responses obtained in anesthetized animals is included in Supplementary Fig. 2. (**f**) Mean movement (mov) traces obtained for each depth of ISMS (*n*≈50 pulses per depth, under 1.5% isoflurane) overlaid on a respective normalized (colour-coded) amplitude map (single animal). Mov onsets and offsets were as graphed (mean±s.d., *n*=9 animals). (**g**) Occurrence, per depth, of mechanical stimulus-evoked MUA responses (light blue) and ISMS-evoked movements (black) per animal (*n*=9). (**h**) Left: Mean stim-evoked MUA frequency (light blue, *n*=15 animals) and ISMS evoked-mov amplitude (black, *n*=9 animals) per depth. Right: Corresponding mean stim-evoked response onsets and ISMS-evoked mov onsets per depth. *P<0.05 (Wilcoxon rank-sum test).

To explore the dynamics of spinal cord network activity in relation to early motor behaviour, we developed a method enabling recording simultaneously translaminar, spinal cord network events and movements, *in vivo*. Subjects were neonatal rats, 5–7 days old (P5–P7, P0=day of birth). Extracellular, local field potential (LFP) and multiunit activity (MUA) were recorded through a linear silicone-based electrode array (16 recording sites with a centre-to-centre separation of 100 μm). Motor behaviour (left hindlimb movements) was recorded using a piezoelectric transducer. Our experimental setup is schematically illustrated in Fig. 1a. The electrode array was inserted at the lumbar enlargement (hindlimb representation), at the L3-L5 left hemisegment level. Anatomical location of each recording site was estimated based on the electrode array insertion coordinates and on the corresponding or age-matched histological assessment (as demonstrated in Fig. 1b and Supplementary Fig. 1). Throughout the text, depth refers to the subdural spinal depth along the electrode array axis.

Systematic mechanical stimulation of different points along the hindlimb allowed establishing topographic relationships, identifying the location of intraspinal sensory neurons and examining the spatiotemporal properties of mechanical stimulus-evoked activity in the ipsilateral L3-5 spinal hemisegment. Mechanical stimulation consisted of a brief (5–10 ms) touch, provided at a rate of 0.2 s^−1^. The two recording sites (or equivalent intraspinal depths) showing the shortest response latencies were considered as sensory zone. Figure 1c shows a case example of response (sensory zone, average within-burst MUA frequency) by stimulation point, reminiscent of the large receptive fields previously described for this age group^36^,^37^. Stimulations of the back, tail, contralateral hindlimb and forelimbs did not produce alterations in spinal activity in L3-5 hemisegments. The stimulation point for which we obtained the shortest latency and highest multiunit frequency response was considered topographic, and this response was analysed further. While mapping was done essentially under light anesthesia (0.5% isoflurane), recordings of topographic, mechanical stimulus-evoked activity were done under no or light anesthesia (0.5% isoflurane). In both conditions, the stimulus reliably evoked a primary, sharp LFP deflection and sink within dorsal horn laminae, with field potentials reversing at relatively deeper dorsal horn laminae (Fig. 1d, non-anesthetized animal, and Supplementary Fig. 2, anesthetized animal—same as in Fig. 1d). These field events were associated with an increase in spiking activity (Fig. 1e), from 11.9±4.6 s^−1^ (baseline) to 193.2±141.4 s^−1^ (average within-burst), (mean±s.d., *n*=10 non-anesthetized animals, *P*≈0.002, Wilcoxon signed-rank test). Evoked bursts onset and offset were estimated at 12±2 ms and 77±43 ms post-stimulus, respectively (mean±s.d., *n*=10 non-anesthetized animals, Fig. 1e). For the vast majority of cases (12 of 14 animals), a secondary activity burst also was observed. These secondary bursts were restricted to the upper most regions activated by mechanical stimulation (generally at the subdural depths of 100–200 μm, consistent with the location of laminae I-II), and occurred with a delay ranging from approximately 70 to 100 ms in relation to stimulus onset. Primary bursts likely result from incoming A-fibre inputs, and secondary bursts might reflect responses to C-fibre activation, as shown previously^36^. While we obtained qualitatively and quantitatively similar sensory responses under no or light anesthesia, one main difference was found: in the no anesthesia condition, sensory bursts correlated with activity in motor zones (Fig. 1e and Supplementary Fig. 2). This activity profile is reminiscent of the activation of reflex pathways. In addition, the mean baseline MUA in sensory zones was approximately twofold higher in the absence of isoflurane: 4.9±2.4 s^−1^ (with isoflurane, *n*=9 animals) and 11.9±4.6 s^−1^ (without isoflurane, *n*=10 animals) (mean±s.d., *P*≈6.5 × 10^−4^, Wilcoxon rank-sum test). The mean MUA in motor zones also was higher in the absence of isoflurane: 9.6±6.3 s^−1^ (with isoflurane, *n*=9 animals) and 18.2±11.4 s^−1^ (without isoflurane, *n*=10 animals) (mean±s.d., *P*≈0.045, Wilcoxon rank-sum test). It should be noted that topographic, mechanical stimulus-evoked burst onsets in sensory zones tended to decrease from P5 to P7 (*n*=15 animals; pooled data with and without isoflurane, *P*=0.0531, Kruskal–Wallis test), about 2 ms per day (Supplementary Fig. 2), which is largely in agreement with previous reports on the development of sensory processing in the spinal cord^37^,^38^ and, in particular, with fibre myelination^39^,^40^.

To probe the anatomical location of premotor/motor networks, we provided brief electrical pulses (50 μs) at different spinal cord depths, with electrical stimulation sites matching recording sites (intraspinal microstimulation, ISMS). For that purpose, a bipolar electrode was lowered to the maximum depth used for recordings (expected location of motoneurons): the minimum current required to elicit detectable movement was determined and used at all depths (100–200 μm steps). Electrical stimulation was performed in deeply anesthetized (1.5% isoflurane) animals. Muscle activity was reliably evoked for electrical stimulations in intermediate to ventral horn laminae (Fig. 1f). Movement onset and offset (as detected on the piezoelectric transducer signal) were located at 11±2 ms and 146±15 ms post-stimulus, accordingly (mean±s.d., *n*=9 animals, Fig. 1f). The two depths of electrical stimulation associated with the highest amplitude movements were considered as motor zone.

Closer examination of the depth distribution of topographic mechanical stimulus-evoked MUA across animals revealed increases in spiking between a minimum depth of 100 μm and a maximum depth of 600 μm (*n*=9 animals, Fig. 1g), with the highest within-burst spiking rates being located at 100–300 μm, estimated to correspond roughly to laminae I-IV (*n*=23–37 responses of 15 animals per depth range, *P*=0.0046, Wilcoxon rank-sum test, Fig. 1h). Consistently, short-latency MUA responses also were found predominantly at the depths of 100–300 μm (*n*=23–37 responses of 15 animals per depth range, *P*=0.0030, Wilcoxon rank-sum test, Fig. 1h). Indeed, while in adult rats Aβ-fibre inputs (innocuous tactile stimulation) are restricted to laminae III-IV, in neonatal rats A-fibre inputs reach superficial and deeper dorsal horn laminae^9^,^38^. Muscle activity, on the other hand, was evoked for stimulations at a minimum depth of 600 μm (deep dorsal horn) and reached the highest magnitude when stimulations were performed at a depth of 1,000–1,200 μm (ventral-most regions, *n*=14–16 responses of 9 animals per depth range, *P*=0.046, Wilcoxon rank-sum test), without any depth-dependence in movement onset (Fig. 1h).

### Network dynamics during twitches and complex movements

**Figure 2:**
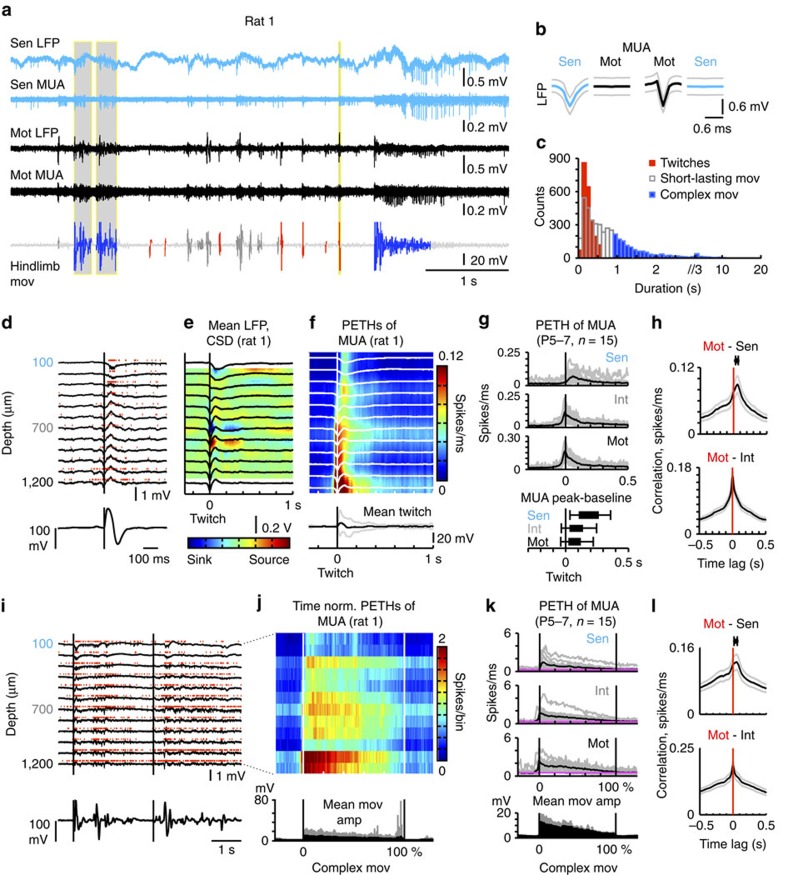
*In vivo* network correlates of spontaneous behaviour. (**a**) Original wide-band LFP signal and MUA (filtered LFP, 300–4,000 Hz) corresponding to one sensory zone (sen) recording site (light-blue traces) and one motor zone (mot) recording site (black traces), and simultaneously recorded hindlimb movements (mov) signal (red: twitches, blue: complex mov, dark grey: short-lasting mov, light grey: baseline). (**b**) Spike waveforms for the two channels included in a (original wide-band LFP signal, mean±s.d., single animal). Left: sen spike-triggered sen and mot LFP. Right: mot spike-triggered mot and sen LFP. (**c**) Histogram of hindlimb movement durations (colour code as in **a**). (**d**) The twitch highlighted in **a**) is shown here in more detail (back vertical line: onset). (**e**) Mean LFP traces and CSD map triggered by twitch onset (*n*=330 twitches, single animal). (**f**) Corresponding normalized peri-event time-histograms (PETHs) of MUA, and mean twitch waveform (black and grey traces: mean±s.d.). (**g**) Top: normalized time-histograms of sen, intermediate zone (int), and mot MUA (black—mean; grey—individual animals; *n*=15 animals). Bottom: Sen, int and mot MUA peak frequency and return to baseline times in relation to twitch onset (mean±s.d., *n*=2217 twitches of 15 animals). (**h**) Top: Normalized cross-correlogram of mot and sen spikes referent to twitching epochs (black and grey—mean±s.e.m., *n*=15 animals); the mean peak lag was 54 ms. Bottom: Equivalent normalized cross-correlogram of mot and int spikes. (**i**) Exemplification of behavioural events classified as complex movements (highlighted **a**; black vertical lines—onsets); (**j**) Normalized time-histograms of MUA triggered by complex movement onset, and complex movement amplitude per bin (black and grey—mean±s.d., *n*=231 complex movements of a single animal). (**k**) Corresponding histograms of MUA for three main zones analyzed, for all animals (*n*=15). (**l**) Top: Normalized cross-correlogram of motor and sensory spikes occurring during complex movement epochs (black and grey traces: mean±s.e.m., *n*=15 animals); the mean peak lag was 41 ms. Bottom: Equivalent normalized cross-correlogram of mot and int spikes.

We recorded spontaneous spinal cord activity and concomitant motor behaviour in non-anaesthetized animals, under buprenorphine analgesia. Spontaneous spinal cord activity was characterized by intermittent LFP deflections and correlated MUA bursts (Fig. 2a,b), which, within sensory and motor zones, occurred at the frequencies of 4.3±1.0 min^−1^ and 3.6±0.9 min^−1^, respectively (mean±s.d., *n*=15 animals, 4249 sensory zone bursts and 3362 motor zone bursts). The occurrence of activity bursts did not differ significantly between the two zones (Wilcoxon rank-sum test). Mean multiunit spiking activity also did not differ significantly between motor (55±20 s^−1^) and sensory (41±18 s^−1^) zones (mean±s.d., *n*=15 animals, Wilcoxon rank-sum test). Temporal analysis of spinal cord activity and ipsilateral hindlimb movements (4.5±2.1 min^−1^, mean±s.d., *n*=15 animals, 4378 events) revealed that ≈75% of the recorded MUA bursts in motor zones were followed (within 100 ms) by overt hindlimb movement. Importantly, the majority (≈60%) of MUA bursts in sensory zones were preceded (within 100 ms) by movement.

Motor behaviour was characterized, qualitatively, by relatively abundant twitching of the extremities, which occurs typically during bouts of active sleep, and attempted crawling and pivoting, which at these ages (P5-7) dominate awake behaviour in rats^41^. Indeed, of analysed independent behavioural events (*n*=4378), 53±15% (mean±s.d.) fell in the twitches category, which was defined essentially as all events lasting less than 600 ms (252±122 ms, mean±s.d.) and occurring in a background of atonia, i.e. in the absence of preceding or following, continued motor activity (Fig. 2c). Complex movements (events lasting more than 900 ms, 1.7±1.4 s, mean±s.d.) were 29% of analysed events (Fig. 2c; for methodological details, see Supplementary Fig. 3). Parallel simultaneous recordings of limb movements and nuchal muscle activity (electromyogram (EMG)) in an additional group of animals (*n*=3) revealed that limb movements occurring during bouts of sleep (twitches/startles) on average last 376 ms (*n*=1,237), and that limb movements occurring during awake periods are in average of 1.3 s duration (*n*=461), and thus complex movement sequences likely represent awake behaviour. Overall, these results are in line the predominance of active sleep and of the twitching behaviour in neonatal rats, over the first postnatal week^42^.

Analysis of translaminar network dynamics during twitching epochs revealed that twitches were reliably preceded by relatively short-lasting bursting activity in intermediate-ventral zones, whereas activity within sensory zones, given by LFP deflections, current sinks and increased firing rates, was consistently observed only following twitch onset (MUA peak at 106±69 ms after twitch onset) (Fig. 2d, single twitch; Fig. 2e,f, single animal; Fig. 2g, *n*=15 animals, 2,217 twitches). The observed general pattern of peri-twitching network activity includes two additional main features. First, in all animals, we found two main current sinks initiated prior to twitching, within the ventral and intermediate spinal cord. Second, increases in spiking frequency within sensory and motor zones overlapped, to some extent, after twitch onset. Cross-correlating motor and sensory spikes occurring within a −0.3 to 1 s time period in relation to twitch onset revealed peak correlations at a lag of 54±21 ms (sensory zone spike times referenced to motor spike times, mean±s.d., 15 animals, Fig. 2h). Cross-correlating spike times in motor and intermediate zones further confirmed a predominantly synchronous activation of these two zones (Fig. 2h).

When analysing spinal cord network dynamics during complex movements, we observed a phenomenon similar to that described for twitching epochs, with activity in intermediate-motor zones preceding complex movement onset, in turn followed by elevated firing of dorsal units, which was maximal after movement onset and declined through the time course of the complex movements (Fig. 2i–l). We did not find fundamental differences in the spatiotemporal organization of network patterns (LFP and MUA) during twitching or complex movement epochs, at the single segment level, except that complex movement-related events lasted longer than those associated with twitches.

### Deafferentation suppresses activity bursts in dorsal horn

**Figure 3:**
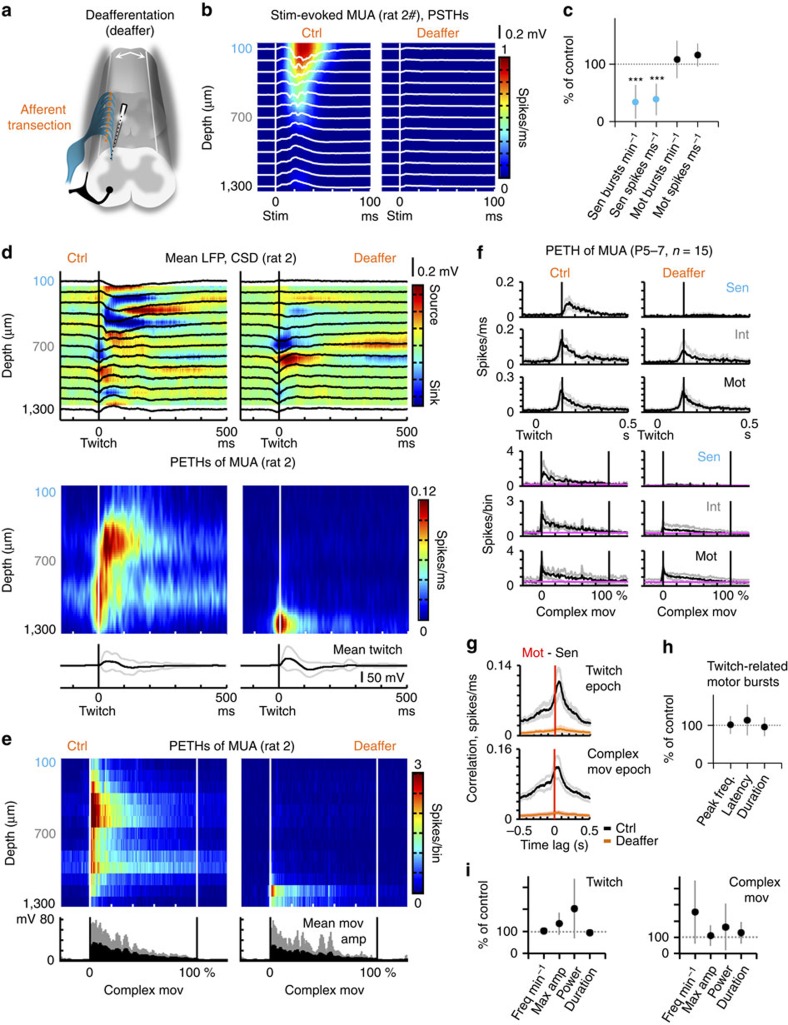
*In vivo* deafferentation suppresses spontaneous dorsal horn bursting. (**a**) Schematic representation of the local deafferentation procedure performed in a subset of animals (*n*=3 of 15). (**b**) Mechanical stimulus (stim)-triggered mean LFP traces and corresponding normalized PSTHs of MUA before (Ctrl) and after deafferentation (Deaffer), (*n*≈100 stimuli per condition, single animal). Note the lack of stim-evoked responses in the after condition. (**c**) Global effect of deafferentation on bursting and mean firing frequencies in sensory (sen, light blue) and motor (mot, black) zones, graphed as percentage of control (mean±s.d., *n*=3 animals). (**d**) Effect of deafferentation on spinal cord network dynamics during twitching (single animal). Top: twitch onset-triggered mean LFP traces and CSD maps before and after deafferentation (n_BeforeDeaffer_=66 and n_AfterDeaffer_=108 twitches). Bottom: Corresponding normalized PETHs of MUA, and mean twitch waveforms (black and grey traces: mean±s.d.). (**e**) Effect of deafferentation on network dynamics during complex movement epochs (single animal). Normalized time-histograms of MUA aligned to complex movement onset (n_BeforeDeaffer_=66 and n_AfterDeaffer_=108 complex movements). (**f**) Top: normalized twitch onset-triggered time-histograms of sen, int and mot MUA before and after deafferentation (black and grey traces: mean±s.d., n_BeforeDeaffer_=273 twitches and n_AfterDeaffer_=321 twitches). Bottom: normalized complex movement onset-triggered histograms of sen, int and mot MUA before and after deafferentation (black and grey traces: mean±s.d., n_BeforeDeaffer_=273 twitches and n_AfterDeaffer_=321 twitches). (**g**) Cross-correlograms of peri-twitching and peri-movement mot and sen spikes before and after deafferentation (black and grey traces: mean±s.e.m.). (**h**) Effect of deafferentation on peri-twitching ventral spiking activity (% of control, mean±s.d.). Graphs showing, for mot, the twitches-related within-bursts MUA peak frequency (spikes ms^−1^) and respective time in relation to twitch onset (latency), as well as bursts offset (peak frequency to baseline return—duration). (**i**) Spontaneous behaviour. Twitches and complex movements frequency (min^−1^), maximal amplitude, power (normalized to duration) and duration after versus before transection of afferent fibers (% of control). ***P<0.001 (a full description of the statistical test used is included in the ‘Materials and methods’ section).

Next, we examined, causally, whether activation of sensory neurons by a feedback mechanism is at the origin of the twitches/complex movement-related dorsal horn activation. For that purpose, in a subset of animals (*n*=3 of 15), we recorded spinal cord activity before and after deafferentation. Specifically, we transected dorsal rootlets innervating the electrode array-implanted region, as illustrated in Fig. 3a (see also Supplementary Fig. 4). Transection was performed in deeply anesthetized (1.5% isoflurane) animals. Local deafferentation was confirmed by lack of topographic, mechanical stimulus-evoked LFP deflections and correlated MUA bursts (Fig. 3b).

Transection of dorsal rootlets caused, in all animals, a striking reduction (of approximately 66±30%, mean±s.d.) in the occurrence of bursts within sensory zones (Fig. 3c). Consistently, the spiking capacity of neuronal populations within the dorsal also was decreased following deafferentation (the mean firing rate in sensory zones after deafferentation—through entire recording sections, per animal—was 39±28% of the control condition, Fig. 3c). Activation of dorsal horn neurons after twitching or complex movements, as observed in the control condition, was strikingly diminished or completely abrogated by deafferentation (Fig. 3d,e, single animal, and Fig. 3f, group data). Consistently, the previously described correlation between spike times in motor and sensory zones during twitching or complex movement epochs was no longer present after deafferentation (Fig. 3g).

The partial overlap between activity in sensory and motor zones raises the possibility that sensory feedback contributes to motor activity. Following afferent transection, activity within motor zones was at least to a large extent preserved (Fig. 3c, group data—general effect, Fig. 3d,e, single animal, and Fig. 3f, group data—different epochs), as we did not detect alterations in bursting frequency (Fig. 3c, group data—general effect), or in the peri-twitching bursting behaviour, including multiunit frequency peak value and time, as well as return to baseline (Fig. 3h). Moreover, the occurrence of twitches, as well individual twitch maximal amplitude, power (that is, signal waveform power normalized to duration) or duration did not change following afferent transection (Fig. 3i). Similarly, global metrics of more complex motor behaviours were not affected by the deafferentiation procedure (Fig. 3i). Following deafferentation, movements were consistently evoked by electrical stimulation at the expected depths (Supplementary Fig. 4), as demonstrated before (Fig. 1).

Together, our *in vivo* data suggest that the predominant function of developing spinal cord networks consists in generating spontaneous bursts in intermediate to ventral motor zones, which drive twitches and complex movements, that both types of movements cause, through sensory feedback, activation of dorsal sensory zones, and that sensory feedback is instrumental for the synchronization of activity in sensory and motor zones of the spinal cord.

### Dissociation of sensory-motor network function *in vitro*

**Figure 4:**
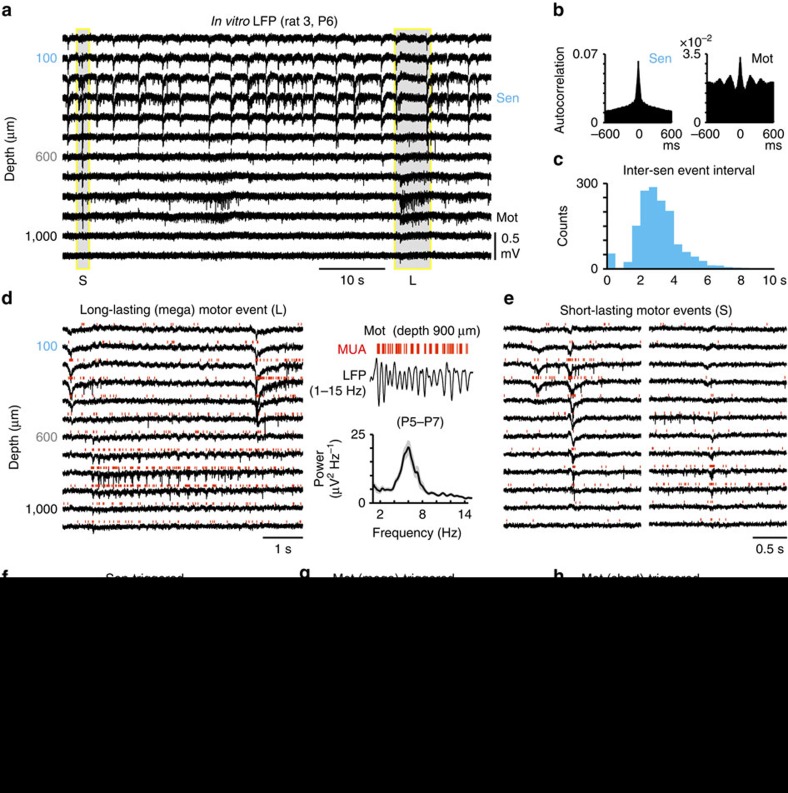
*In vitro* spontaneous patterns of activity. (**a**) Original wide-band LFP traces representing stereotypical sensory zone (sen) bursts and heterogeneous motor zone (mot) events (S: short-lasting mot event; L: long-lasting mot even). (**b**) Autocorrelograms of spikes in selected sen and mot recording sites. (**c**) Histogram of inter-sensory event intervals (with a peak at 3 s). (**d**) Left: original wide-band LFP traces and MUA (red) across all spinal depths; the long-lasting mot event indicated in (**a**) is shown here in more detail. Top right: filtered LFP (1–15 Hz) trace and coherent MUA at a recording depth of 900 μm. Bottom right: Mean power spectrum of bursts referent to a selected mot recording site (mean±s.d., *n*≈760 bursts of 5 animals). (**e**) Exemplification of short-lasting mot events. Original wide-band LFP traces and MUA (red). (**f**) Sensory events-triggered mean LFP and CSD maps (single animal). (**g**,**h**) Equivalent analysis, with long-lasting or short-lasting motor events as triggers, respectively.

**Figure 5:**
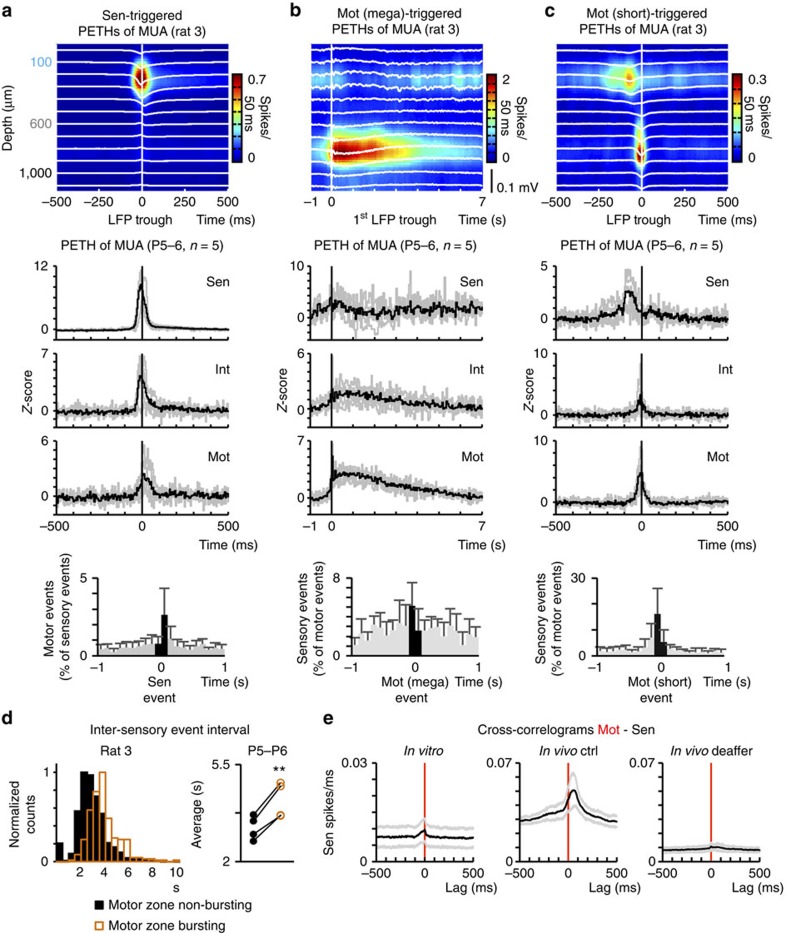
Dissociation of ongoing dorsal and ventral activities *in vitro*. (**a**) Top: Sensory (sen)-bursts triggered normalized histograms of MUA across all spinal depths (single animal). Middle: corresponding normalized PETHs of MUA of sensory (Sen), intermediate (Int) and motor (Mot) zones (Z-scores, grey traces: single animals; black trace: mean, *n*=5 animals). Bottom: percentage of detected surrounding motor events (mean±s.d., *n*=5 animals). (**b**,**c**) Equivalent graphics instead triggered by long- and short-lasting motor events, respectively. (**d**) Left: Representative histograms of inter-sensory event intervals during non-bursting and bursting motor zone periods (black and orange, respectively, single animal). Right: Mean inter-event interval values during non-bursting and bursting motor zone periods per animal (*n*=4). (**e**) Cross-correlograms of all mot and sen units recorded per animal *in vitro* (mean±s.e.m., *n*=5 animals), *in vivo* under control conditions (mean±s.e.m., *n*=15 animals) and *in vivo* after deafferentation (mean±s.e.m., *n*=3 animals). **P<0.01 (paired two-tailed Student’s *t*-test).

Spontaneous activity also has been described *in vitro*, using the neonatal rat spinal cord preparation or neonatal rat spinal cord slices, in which correlated sensorimotor activity may be supported by intrinsic connections and emphatic interactions^33^,^34^,^35^,^43^. Therefore, we further investigated interactions between sensory and motor zones in the *in vitro* spinal cord (from P5-7 rats) preparation, through silicone probe recordings, as performed *in vivo* (Supplementary Fig. 5). Spontaneous activity in isolated spinal cords was characterized by regular bursts within the dorsal horn, occurring at 15±4.9 min^−1^ (mean±s.d., *n*=5 animals, 6860 bursts) (Fig. 4a,c). These were associated with a dorsal sink (Fig. 4f) and MUA bursts (Fig. 5a), lasting approximately 119±28 ms (mean±s.d., *n*=5 animals, 6,860 bursts). A main feature of activity in motor zones was its organization in megabursts−3.0±0.3 s long periods of oscillatory activity at 6.0±0.1 s Hz, occurring at 1.7±0.3 min^−1^ (mean±s.d., *n*=5 animals, ≈760 bursts; Fig. 4d,g). These megabursts could be also evoked by rhythmic dorsal root stimulation (Supplementary Fig. 5). Isolated, short lasting bursts (with a duration of about 62±14 ms), reminiscent of twitch-related events *in vivo*, also occurred in motor zones, at a frequency of approximately 2 min^−1^ (5 animals, 760 bursts; Fig. 4e,h).

Sensory or motor bursts-triggered histograms of MUA, and cross-correlation analysis of bursts in both zones revealed weakly (or failed to reveal any) correlated firing (Fig. 5a–e). Indeed, only about 3% of bursts within sensory zones were followed (within a maximum lag of 100 ms) by bursts within motor zones (average, *n*=5 animals, for which in total 6860 events were detected). Moreover, in average, only 5% of mega- and 16% of short-lasting motor bursts were preceded by sensory bursts. Further cross-correlation analysis of units in sensory and motor zones referenced to entire recording sessions showed only a weak correlation, with sensory neurons firing 2–46 ms ahead of motor units (peak value of 5.4 spikes s^−1^). This is in striking contrast to the activity recorded *in vivo*, where motor units led sensory activity both during twitching and complex movement episodes, with a lag of 58±22 ms (peak value of 24±17 spikes ms^−1^, mean±s.d., *n*=15 animals). It also differs from the overall activity pattern through entire recording sessions after transecting dorsal rootlets *in vivo*, where the correlation between the two zones was dramatically decreased or completely lost (Fig. 5e).

Pharmacological analysis of spontaneous activity in the isolated spinal cord revealed that combined application of the ionotropic glutamate receptor antagonists CNQX and APV completely suppressed network-driven events both in sensory and motor zones (Supplementary Fig. 6), and that blockade of GABA(A) receptors with gabazine eliminated regular bursts in sensory zones and significantly increased LFP power and MUA during motor megabursts, with loss of the prominent within-burst 6 Hz oscillation; during these epileptiform discharges in motor zones evoked by disinhibition^44^, activation of neurons in sensory zones was not observed (Supplementary Fig. 7).

Thus, in contrast to the *in vivo* situation, activity in the isolated spinal cord was characterized by much weaker interactions between sensory and motor zones, and by a temporal leading of sensory neurons in the isolated sensory-motor network, inverse to the *in vivo* situation, where motor zones headed population activity. Taken together, data obtained in the isolated spinal cord provide strong support to the hypothesis that sensorimotor integration in spinal cord under physiological conditions is primarily driven by sensory feedback resulting from motor unit-driven movements.

## Discussion

We explored the spatiotemporal dynamics in spinal cord sensorimotor networks in relation to spontaneous movements of neonatal rat pups *in vivo*. We observed highly correlated activity in motor and sensory zones of the spinal cord during spontaneous movements, both twitches and complex movements, with the following activation sequence: motoneurons—movements—sensory neurons. Deafferentation through local transection of dorsal roots/rootlets profoundly suppressed activity bursts in sensory zones and desynchronized sensory and motoneurons without affecting global metrics of activity in motor zones and movements. Dissociation of the activity in motor and sensory zones also was observed in the isolated spinal cord preparation *in vitro*. Taken together, our results strongly support the hypothesis that sensory feedback from spontaneous movements is instrumental for the temporal binding of motor and sensory neurons in the developing spinal cord.

The hypothesis that sensory feedback, or reafferentation, synchronizes the activity of motor and sensory neurons during neonatal spontaneous movements is based on the assumption that movements are associated with the activation of proprioceptors, tactile and eventually pain receptors. Provided that primary afferents and sensory neurons are mature enough, activation of primary sensory neurons during movements should result in activation of secondary sensory neurons in the spinal cord. This hypothesis is supported by a number of observations. First, sensory inputs drive activity in the spinal cord dorsal horn from early developmental stages^38^. Second, conditioning through artificial introduction of *aberrant* sensory input during twitching leads to an increased nociceptive reflex error rate, thus underlying sensory-to-motor connectivity might be guided by sensory feedback^7^,^9^. Finally, spontaneous twitches reliably precede activation of thalamo-cortical networks^11^,^13^,^45^. The detailed characterization of the translaminar spinal network dynamics in behaving rat pups provided by this study further supports the sensory feedback hypothesis. We observed that activation of sensory neurons follows twitch/complex movement onset, and that the delay of MUA bursts in sensory zones from movement onset is close to the delay of sensory-evoked responses. In the case of an underlying excitatory efferent copy mechanism, as suggested on the basis of results obtained in the isolated spinal cord *in vitro*, the delay in sensory neuron activation should have been much shorter, or sensory neuron activation could even have preceded movement onset^34^. Moreover, we found that local deafferentation almost completely eliminated activation of sensory neurons during spontaneous movements. This directly indicates that sensory feedback is a necessary condition for correlated activation of motor and sensory neurons. Finally, we also failed to observe any prominent correlated activity between sensory and motor neurons in the isolated spinal cord preparation *in vitro*, despite the rich repertoire of spontaneous activity patterns occurring essentially independently in motor and sensory zones. Previous studies revealed that different types of neonatal spontaneous movements, twitches and complex movements, evoke cardinally different cortical responses^29^,^30^. Twitches and startles, occurring typically during active sleep, are followed by excitation of neurons in primary motor cortex and hippocampus, which is largely in agreement with the results that have been obtained in the present study. In contrast, complex, long-lasting movements that occur during awake periods failed to activate cortical regions. It has been suggested that these two types of motor events are processed differently, the twitches being processed as unexpected events, whereas complex movements are associated with inhibition of cortical activation via corollary discharges. Gating of sensory inputs during complex movements could occur at the level of spinal cord, for example, via local GABAergic interneuron-mediated inhibitory corollary discharge on dorsal sensory neurons and primary afferents^32^. In the present study, we found that complex movements are associated with the activation of neurons in sensory zones (Fig. 3), pointing to supraspinal mechanisms of inhibition of cortical-hippocampal activation during complex movements.

Interestingly, activity in motor spinal cord regions and corresponding twitches and complex movements were not grossly affected by deafferentation, which is in keeping with previous behavioural results, where no effect of deafferentation on the expression and structure of twitches was shown^7^. This is in apparent contradiction with our findings that sensory stimuli activate not only sensory, but also intermediate and motor units (Fig. 1), suggesting that sensory feedback has the potential to modulate motor bursts through the excitatory feedback loop involving sensory feedback from movements. However, neither firing of motor units nor duration of motor bursts associated with twitching or complex movements (Fig. 3) were reduced by deafferentation, indicating the autonomy and robustness of the motor generating circuits. It should be noted that, both in the present and in previous studies^7^, spinal motor activity and associated spontaneous movements were assessed only during a relatively short period of time after deafferentation. However, changes in the normal expression and development of motor activity and behaviour are likely to occur at a longer time scale^25^, in line with the model of plasticity induced by sensory feedback in developing sensorimotor networks^9^.

Our study revealed the temporal organization and intralaminar dynamics of sensory-evoked responses in the neonatal rat spinal cord *in vivo*, at the network level. We found that there are important age-specific differences in the properties of sensory-evoked responses in neonatal rats compared to adult animals. First, the response delays were longer, which likely reflects the myelination process^39^,^40^,^46^,^47^ and matches the delays reported previously^36^,^37^. In agreement with previous results, we also observed a much shorter delay between the responses conveyed by tactile and putative nociceptive afferents. Putative C-fibre inputs triggered spiking of sensory units located exclusively in superficial laminae and, in the no-anesthesia condition, also triggered spiking of motor units, likely reflecting the activation of the nociceptive withdrawal reflex networks^9^,^38^. Second, while in adult animals sensory stimulation leads to short-lasting responses, we found that in P5-7 rats, sensory stimulation evokes relatively long-lasting bursts of activity (≈70 ms) in sensory zones. This suggests that the sensory spinal cord network in P5-7 rats operates in an immature bursting mode, as do so many other neuronal networks in that period^19^,^48^,^49^. These relatively long-lasting sensory responses are further conveyed bottom-up and likely contribute to bursting in thalamus-cortex. However, the duration of thalamocortical oscillations evoked by sensory stimulation is significantly longer, up to 1 s^13^,^14^. In addition, sensory-evoked bursts in the spinal cord were not organized in any prominent oscillatory pattern in the frequency range characteristic of EGOs^11^,^14^,^28^ or spindle-bursts^11^,^13^,^50^. This suggests that sensory output from the spinal cord triggers, but does not drive thalamocortical oscillations. This is also supported by the findings that spindle-bursts in S1 hindlimb area persist, although at lower frequency, after spinalization at mid-thoracic level, that completely eliminates input from the secondary sensory neurons^13^.

Our main goal of using the isolated spinal cord preparation was to explore whether and how local intraspinal circuits directly support interactions between sensory and motor neurons, independently of sensory feedback, during bursting network behaviours. In keeping with the results of previous studies, the *in vitro* neonatal rat spinal cord preparation displayed spontaneous bursting activity^33^,^34^,^35^,^44^. Interestingly, the activity in motor zones was organized in two main patterns: short-duration bursts, which are reminiscent of the short twitch-producing bursts *in vivo* (see also Bos *et al*.^34^), and long duration megabursts, which are similar to the *in vivo* complex motor events. The *in vitro* megabursts were characterized by prominent 6 Hz oscillations, which were never observed during complex bursts *in vivo*, however. Such spontaneous complex movement-like events, which typically occur during awake, explorative states, have not been reported previously with respect to the isolated spinal cord. This finding indicates that not only twitches, but also complex motor events can be generated in the neonatal spinal cord independently of top-down commands. In the dorsal horn, the activity was organized in regular short bursts, similar in duration to the twitch-triggered bursts, yet occurring at much higher frequency than *in vivo*, particularly in the deafferentated state. Thus, despite of some difference in the frequency of events and oscillatory feature of the megabursts, the global organization of activity in the isolated spinal cord *in vitro* was remarkably similar to the activity patterns expressed by the spinal cord *in vivo*, considering the activity in dorsal and ventral zones separately. However, a striking difference was found in the temporal correlations between the activity in motor and sensory zones, which essentially behaved independently, as in the deafferentated spinal cord *in vivo*, and the mere correlations, when expressed, were inverted from a motor to sensory lead *in vivo* to a sensory to motor lead *in vitro* (see also Bos *et al*.^34^). These results confirm our conclusion on the instrumental role of sensory feedback in synchronizing sensory and motor neurons, as revealed by our *in vivo* experiments, while pointing to limited roles of local communication between motor and sensory neurons in the generation of coordinated sensorimotor activities.

Pharmacological analysis indicated a primary contribution of glutamatergic mechanisms in the generation of spontaneous bursts in motor and sensory zones, as all organized activity was suppressed by glutamate receptor antagonists, in agreement with previous studies^34^,^51^,^52^. However, GABAergic mechanisms differently contributed to the function of dorsal and ventral networks: while the recurrent bursts in dorsal horn were completely suppressed by the GABA(A) receptor antagonist, activity in motor zones was boosted and converted to epileptiform discharges (see also refs 34, 44, 53). Importantly, even these hypersynchronous events in the disinhibited motor zones failed to activate dorsal horn neurons, indicating that glutamatergic networks implicated in generation of motor activity do not project to dorsal neurons. This is also in keeping with a perinatal switch in GABA actions from excitatory to inhibitory on motor and sensory spinal cord neurons^54^,^55^,^56^,^57^,^58^. On the other hand, spontaneous bursts in dorsal roots and following them excitatory postsynaptic potentials in motoneurons and ventral root discharges in the spinal cord *in vitro* are suppressed by the NKCC1 blocker bumetanide and enhanced by the positive allosteric GABA(A) modulator diazepam, indicating an involvement of NKCC1-dependent excitatory actions of GABA on primary afferents^34^. Yet, in the intact neonatal animals *in vivo* bumetanide was shown to have little effect, whereas diazepam was shown to exert mainly inhibitory actions on spontaneous and sensory-evoked activity in the somatosensory and visual cortex suggesting limited contribution of the NKCC1-dependent excitatory networks actions of GABA *in vivo*^59^,^60^,^61^. Such a difference in the GABAergic contributions to the spinal cord functions could explain the inversed temporal correlations between the activity in sensory and motor zones and higher frequency of the dorsal bursts *in vitro* than *in vivo*, but this remains to be experimentally demonstrated.

The main goal of our study was to characterize the dynamics of motor and sensory networks in the spinal cord of neonatal rats in the context of their motor behaviour. Our main conclusion is that the activity in sensory zones follows the activity in motor zones, and that sensory feedback resulting both from short twitches and more complex movement sequences is instrumental for sensorimotor synchronization. While the idea of the importance of sensory feedback from neonatal movements in driving the activity in sensory networks is not novel, the present study provides strong evidence to this hypothesis through an exploration of the dynamics of sensorimotor networks in the developing spinal cord.

## Methods

All experiments were performed in accordance with protocols approved by the Ethical Committee for Animal Research of Marseille (reference 00708.01).

### Subjects

Subjects, in total 25, were 5–7 days old male and female Wistar rats, weighing 12.0–22.6 g, from multiple litters. Animals were obtained from the in-house breeding colonies. All animals were housed in enriched laboratory cages, in climate-controlled rooms (22 °C, 60% humidity), under a 12 h light/12 h dark cycle and with *ad libitum* access to water and food. Prior to the onset of experiments, we verified that the pups had been recently fed (with ingested milk being visible through the abdominal skin). Animals were randomly selected from multiple litters; age selection was based on colony availability. Experimental cases that did not meet the pre-established criteria—first, the positioning of the electrode, which was verified *post hoc*, and, second, lack of discernable tissue damage—were excluded from the study (*n*=2 animals—1 *in vivo*; 1 *in vitro*).

### Electrophysiological recordings *in vivo*

We developed a method for simultaneously recording electrical activity across spinal cord laminae and motor behaviour in neonatal rats, based in part on previous preparations for chronic imaging of the dorsal spinal cord and spinal single-wire or patch-clamp electrophysiological recordings in anesthetized, adult rodents^62^,^63^,^64^,^65^. Specifically, prior to and during surgery, animals (*n*=15) were anesthetized using isoflurane (Aerrane) and given the analgesic buprenorphine (0.03 mg Kg^−1^ of body weight, Buprecare). Local anesthesia and vasoconstriction were additionally provided by subcutaneous injection of lidocaine-adrenaline (Xylocaïne-Adrenaline, AstraZeneca) prior to skin incision. Throughout the experiments, body temperature was kept at 37 °C using a heating pad (TC-344B, Warner Instruments). Animals were, at all times, surrounded by a cotton nest, mimicking the presence of the mother and littermates. Dorsal laminectomy was performed typically at the level of vertebrae Th13 and L1. The spinal cord was protected by silicone oil up to the insertion of the electrode array, and a ring-shaped metal adaptor was fixed onto additionally exposed spinal vertebrae, using a thin layer of cyanoacrylate and dental adhesive. The adaptor was attached to a customized stereotactic frame, enabling spinal cord stabilization. The position of the heating path was carefully adjusted, on the z axis, to not constrain natural respiration-related displacements, which could otherwise translate into an upward pressure and spinal displacement. To avoid relatively large movements, animals were partially head-restrained through a thin cylinder-shaped stereotaxic frame adaptor gently fixed onto a small portion of the head. To further avoid potential spinal displacements during tail movements, the tail was very mildly restrained using a small piece of adhesive tape. Buprenorphine was re-administered every 6 h (if applicable).

A linear silicone-based electrode array (15 μm thick, width of 33 μm at the penetrating electrode and of 125 μm at the surface-most electrode; 16 recording sites, with a centre-to-centre separation of 100 μm, each with an area of 703 μm^2^; A1 × 16-5 mm-100-703-A16, Neuronexus) was inserted in the spinal cord. The tip of the electrode array was positioned at 0.3–0.5 mm lateral to the central vein, and the array was lowered, following dura puncture, with a medial to lateral angle of approximately 17°. Labelling of the electrode array with the fluorescent dye 1,1′-Dioctadecyl-3,3,3′,3′-tetramethylindocarbocyanine perchlorate (DiI, Sigma-Aldrich) prior to insertion facilitated the later anatomical location of the array track (Fig. 1 and Supplementary Fig. 1). Spinal pulsations were minimized by topical application of low-melt agarose. Two chlorided silver wires placed at the surface of the spinal cord and vertebral column served as reference and ground electrodes, respectively.

Recordings were initiated after a minimum waiting period of 30 min following implantation of the electrode array and performed as described previously^13^. Briefly, wideband neurophysiological signals were amplified × 1,000 through a custom-built amplifier and acquired at 10 kHz (Digidata, Axon Instruments). Tactile stimuli were provided through a 0.6 mm-diameter metal bar driven by a piezoelectric bending actuator (PAB-4010, Nihon Ceratec), which was controlled by a square pulse (5–10 ms) delivered at 0.2 s^−1^ using a stimulator (Master 8, AMPI) and stimulus isolator (ISO-Flex, AMPI). We performed approximately 100 trials per stimulation point. Spontaneous activity was recorded 30–60 min after discontinuing anesthesia.

Following recordings, animals were given an overdose of urethane (3 g Kg^−1^, Sigma-Aldrich) and perfused intracardially with saline for 1 min and, subsequently, with 4% formaldehyde for 2 min (at a rate of 5 ml min^−1^).

### Motor behaviour

Ipsilateral hindlimb movements were simultaneously recorded through a piezoelectric transducer (KPEG165, Kingstate, Fig. 1a), which enabled detection of the smallest visible movements, including respiration-related ones. The signal was digitized at 10 kHz, as described before (Digidata interface).

### Intra-spinal microstimulation (ISMS)

A bipolar 50 μm nichrome-wire (A-M Systems) electrode was inserted in the spinal cord, under deep anesthesia (1.5% isoflurane). The electrode was lowered to the maximum depth used for recordings (near motoneuron pools); the current required to evoke detectable movements was determined (threshold) and used for all subsequent stimulations. Stimulation sites matched recording sites. Electrical pulses (50 μs, delivered at a frequency of 0.2 s^−1^) at near-threshold current were generated by an AMPI stimulator and stimulus isolator, as above described. We performed approximately 50 trials per stimulation depth.

### Transection of dorsal rootlets

In a subset of animals (*n*=3 of 15), deeply anesthetized using 1.5% isoflurane, the electrode array was retracted from the spinal cord, and durotomy was performed to expose the dorsal rootlets innervating the array-implanted and adjacent regions (Fig. 5a and Supplementary Fig. 4). Rootlets were gently set aside with the aid of an ultrafine forceps and transected using ultrafine micro-scissors. The completeness of local afferent transection was verified by lack of tactile stimulus-induced spinal responses.

### Defining sleep-awake states and related movements

In a separate group of animals (*n*=3 sham, head-restrained animals), we detected limb movements through piezoelectric transducers, as above described, and simultaneously recorded nuchal EMG, essentially as described by others^66^. Signals were amplified and digitized at 32 kHz using a Digital Lynx SX, Neuralynx data acquisition system. Defining sleep-awake states in neonatal rats, at stages in which the expression of characteristic sleep-wake neurophysiological patterns is not yet present is commonly achieved through nuchal EMG recordings^1^.

### Extracellular recordings *in vitro*

Spinal cords were isolated essentially as described previously by others^34^. Animals (*n*=6) were anesthetized using isoflurane, decapitated and rapidly eviscerated. The preparation was immersed quickly in ice-cold, carbogenated (95% O_2_–5% CO_2_) artificial cerebrospinal fluid (aCSF) and pinned down. We then performed a dorsal laminectomy, from sacral to cervical segments. The dura matter was carefully excised, and the spinal cord, together with dorsal and ventral roots, was quickly transferred to and maintained in a holding chamber (containing carbogenated aCSF) at room temperature (RT) for at least 1 h. The spinal cord was then placed, dorsal side up, in a modified interface recording chamber^67^, where it was submerged in and perfused with carbogenated (95% O_2_–5% CO_2_) aCSF (3–4 ml min^−1^) of the following composition, in mM: 130 NaCL, 4 KCl, 2 CaCl_2_, 1.3 MgSO_4_, 0.58 NaHPO_4_, 25 NaHCO_3_ and 10 glucose at RT. The electrode array and implantation procedure were as described for *in vivo* recordings (Supplementary Fig. 5a).

Signals were amplified using a xCellAmp64 amplifier and digitized at 10 kHz (DIPSI). Dorsal and ventral roots were stimulated using a bipolar 50 μm nichrome-wire electrode; electrical pulses (50 μs) were generated by a Grass Products stimulator and stimulus isolator and delivered at 0.2–2 Hz. In a subset of experiments, recordings were performed under control conditions and in the presence of 2R)-amino-5-phosphonopentanoate (APV, 50 μM) and 6-cyano-7-nitroquinoxaline-2,3-dione (CNQX, 20 μM), or 4-[6-imino-3-(4-methoxyphenyl)pyridazin-1-yl] butanoic acid hydrobromide (gabazine, 5 μM), all from Sigma-Aldrich. Upon completion of the experiment, one preparation was gently turned ventral side up, and the silicone probe inserted in order to match the previous dorsal side up configuration; the activity patterns obtained in the dorsal side up preparation were verified in the inversed set-up.

### Histology

Isolated spinal cords were kept in 4% formaldehyde for at least 1 week, at 4 °C. First, to confirm the spinal cord segment in which the electrode array was inserted, we studied the position of the dorsal DiI signal in relation to lumbar ventral roots. In parallel, the (dorsal) lumbar enlargement was imaged: epifluorescence (DiI) and dark-field micrographs were acquired under standardized conditions using an Olympus SZX16 microscope and the CellSens software (Olympus). In acquired images, the area corresponding to the lumbar enlargement (dorsal surface) was divided in six equal segments, considered as L1-L6 (≈1 mm). Next, the lumbar spinal cord was sliced transversally. Slices (200 μm, thick) were imaged as above described prior to immunohistochemistry. We performed choline acetyltransferase (ChAT) and neuronal nuclei (NeuN) immunohistochemistry. Slices (free-floating) were rinsed three times with PBS and kept in a 5% blocking solution—5% normal serum (Jackson ImmunoResearch) and 0.25% Triton X-100 (Sigma-Aldrich) in PBS—for 1 h at RT. Thereafter, slices were incubated with a goat anti-ChAT antibody (1:100, Chemicon, Merck Millipore) and a mouse anti-NeuN antibody (1:200, Chemicon) in 2% blocking solution overnight at 4 °C. After rinsing, slices were incubated with an anti-goat Alexa633-conjugate antibody and an anti-mouse Alexa488-conjugate antibody (both from Molecular Probes, Invitrogen) in 2% blocking solution for 2 h at RT. Fluorescent dyes (DiI, Alexa488 and Alexa633) were imaged using a Leica TCS SP5 X confocal miscroscope (Leica). Note that the DiI signal was strikingly diminished by the immunohistochemistry protocol. To our knowledge, there is no available systematic study on the anatomical organization of the neonatal rat spinal cord. Hence, laminae borders were extrapolated from analysis of dark-field images (slice morphology), ChAT and NeuN immunoreactivity patterns, and knowledge of the adult rat spinal cord. Note that images were linearly altered for better visualization of slice morphology. The anatomical location of each recording site of the electrode array was estimated using the insertion coordinates and the corresponding or age-matched histological assessment (as demonstrated in Fig. 1b and Supplementary Fig. 1). Throughout the manuscript, depth refers to the subdural depth considering the electrode array (and not the dorsal-ventral) axis, for simplification.

### Data analysis

Data were analysed offline using the Axon Instruments software, as well as conventional and custom-made MATLAB routines. Raw data were preprocessed using a custom-developed set of programs in the MATLAB analysis environment. For spike detection, the original wide-band signal was band-pass filtered (300–4000 Hz), and negative events exceeding 3.2 (*in vitro*) to 4 (*in vivo*) × minimum s.d. (using 10 ms windows) were considered spikes. Simultaneous events, that is, spikes detected in more than half of the recording sites with zero jitter, were considered artifacts and eliminated (rare). With respect to *in vitro* recordings, electrical stimulation artefacts were eliminated by truncating the original wide-band signal at −1 to 1 ms considering the stimulus first peak. Positive polarity is depicted up in all figures. Tactile stimulus times or behavioural event onsets (*in vivo*), as well as electrical stimulation of dorsal and ventral roots or spontaneous sensory and motor bursts onsets (*in vitro*) were used as triggers to compute mean LFP and current source density (CSD, second spatial derivative of LFP) maps, as well as individual trigger spectrograms of field. CSD analysis was used to eliminate volume conduction and localize synaptic currents; CSD was computed for each recording site and smoothed with a triangular kernel of length 3. Spectral analysis was carried out using the Chronux toolbox. Spectral power was given by direct multi-taper estimators (generally, 5 Hz bandwidth, 3 tapers, 200 ms spectral window); to remove the slow frequency envelope, the LFP was filtered (5–100 Hz). For presentation, translaminar peri-stim time-histograms (PSTHs) and peri-event time-histograms (PETHs) of MUA, firing rates for each recording site (bin size=1 ms) were smoothed by a 10-point moving average, and a between channels interpolation of 7th order was used.

For each animal and tactile stimulation point (*in vivo*), PSTHs of MUA were computed. Detection of channels showing stimulus-evoked activity was achieved by the Otsu’s method. In all cases, the identified channels were located within the dorsal horn, as estimated histologically. It should be noted, however, that when recordings were done in the absence of isoflurane, an elevation of MUA at the level of the ventral horn also could be found (see Fig. 2 and Supplementary Fig. 2). For each identified channel, the precise average response onset and offset were determined using a trapezoidal sum-based method (adapted from Mitrukhina *et al*.^68^). Then, we selected the two channels showing the earliest onsets; these were always contiguous and the respective depths referred to as ‘sensory zone’. The corresponding within-response MUA frequencies were averaged, and normalized to the maximum value obtained across stimulation points (for each animal). Normalized MUA frequency values were represented as colour-coded dots overlaid on respective stimulation points in aged-matched hindlimb photographs, allowing the creation of a ‘response map’. The stimulation point associated with the shortest onset and maximal MUA frequency (‘topographic’) was considered for further single animal and group data analyses.

An equivalent analysis was carried out with respect to the activity evoked by electrical stimulation of different dorsal roots (*in vitro*).

For each intraspinal depth of electrical stimulation (*in vivo*), the stimulus onset was used as trigger for averaging the corresponding movement signal (recorded through a piezoelectric transducer). Movement onset and offset were determined as described above^68^. Results are presented as the mean signal per depth overlaid on a colour-coded amplitude map, which was normalized to the maximum value obtained across depths of stimulation. The two depths associated with the highest evoked-movement amplitudes were considered as motor zone and used for subsequent analysis. With respect to the intermediate zone, we considered the two recording sites located 300 and 200 μm dorsal to the dorsal-most recording site corresponding to the motor zone; thus, intermediate zones corresponded to the average depths of 680–780 μm.

With respect to activity evoked by electrical stimulation of ventral roots (*in vitro)*, the stimulus onset was used as a trigger for averaging LFP and computing a CSD map. Response-associated recording sites, generally 2, were identified by analysis of PSTHs of MUA and confirmed by visual inspection of single responses (population spikes).

To quantify the relationship between behaviour and spinal cord activity (*in vivo*), we first defined behavioural events. We determined the minimum standard deviation of the original movement signal found in sliding windows of 1–10^6^ ms (log scale), with 50% overlap. For all records, a striking increase in the minimum s.d. was obtained for windows bigger than 10^4^ ms, and thus, a multiplier (generally 4–6) of the minimum s.d. obtained when using 10 ms windows used was as (±) threshold to discriminate noise from real events, on demeaned traces. Then, local minimum and maximum signal values were calculated, and peaks less than 300 ms apart were considered part of a single event, taking into account signal resolution and previous studies^13^,^69^. The first derivative of the demeaned trace was then calculated, and the precise twitch onset given by the first zero of the differential preceding the event’s first intersection with the threshold. Offset detection followed an equivalent rule (Supplementary Fig. 3). Thereafter, events shorter than 600 ms were considered as putative twitches and manually classified using a customized data viewer, showing single cases in a time window of 4 s. Specifically, events were classified as twitches (likely including indiscernible bouts of twitches at different joints) if occurring on a background of atonia (that is, there were no detected events in the preceding and following ≈1 s). All remaining events were classified as short-lasting movements (typically ending complex movement sequences, 26.6% of all detected events) or noise (14.8% of all detected events), and were not considered for further analysis. Events lasting 600–900 ms were considered intermediate behavioural events (and were included in the total number of detected independent behavioural events; note that in Fig. 1a,c, these events also are represented in grey, together with short-lasting movements, for simplification). Events lasting more than 900 ms were considered as complex movements. For the parallel analysis of simultaneous recordings of limb movements (through piezoelectric transducers) and nuchal EMG, piezoelectric transducer-derived and EMG signals were treated independently. Limb movements were detected as above described. With respect to nuchal EMG, the original wide-band signal was filtered (300–700 Hz), demeaned, and negative events exceeding 3.2–4 × minimum s.d. (using 10 ms windows) were detected and transferred to the frequency domain (through 25 ms sliding windows, with a 1 ms overlap). Then, events larger than 1 × s.d. of the frequency trace were detected and joined when: (1) occurring less than 800 ms apart; (2) the mean inter-event activity (original trace, absolute values) was higher than threshold. Finally, events lasting more than 1 s were considered as awake bouts. All remaining periods of the recording session were considered as sleep.

For each animal, translaminar PETHs of MUA, with detected twitch onsets as reference point, were computed essentially as described before. For further analysis of spontaneous translaminar cord MUA, we used data from previously identified sensory and motor zones, as well as from intermediate zones. For each twitch, the mean baseline (entire session) was used to detect MUA frequency peaks in motor, intermediate and sensory zones; results are presented as average MUA frequency peak times and baseline return times. A similar analysis was carried out for complex movements. However, due to the large repertoire of complex movement durations and lack of stereotypy, each complex movement period (onset-offset) was divided in 100 bins, and thus firing dynamics during complex movement epochs are presented as time-normalized PETHs. With respect to pre-onset and post-offset periods, we considered −0.5 and +0.5 s periods, respectively; binning was done considering the mean complex movement duration (1.7 s). For each animal, the mean of pre-onset period MUA activity was considered as baseline mean. Cross-correlation analyses between motor and sensory zone spikes, and between motor and intermediate zone spikes were performed taking into account twitching epochs (300 ms prior to and to 1 s after twitch or 300 ms prior to 1.5 s), complex movement epochs (onset to offset), or entire recording sessions (per animal). Each cross-correlogram (bin size=1 ms) was smoothed by a 3-point moving average.

To further understand temporal correlations between behaviour and spinal cord network activity, we detected activity bursts within sensory and motor zones, based on a previously described method^70^. For that purpose, individual channel’s instantaneous firing rates were calculated using sliding windows of 50 ms (10% overlap). Bursts were generally considered as events exceeding 5 s.d. of the instantaneous firing rate, and events (MUA frequency peaks) less than 500 ms apart were considered as one. Then, event onsets and offsets were estimated taking into account the nearest intersections with the baseline mean (respectively).

To detect *in vitro* bursts of activity within identified sensory and motor zones, we used an approach similar to that described for the *in vivo* data, with a few modifications. Bursts were generally considered as events exceeding 3 s.d. of the instantaneous firing rate. Bursts within sensory zones were rather stereotypical. In motor zones, however, we observed two main event types: short- and long-lasting (or mega-) bursts organized in ≈6 Hz LFP and MUA coherent oscillations. Here, a first threshold (3 s.d.) was used to more sensibly establish mega-bursts, while a second threshold (4 s.d.) was used to eliminate between-bursts potentially spurious increases in firing rates (that is, isolated short-lasting events −56% of detected events were excluded). Here, local minimum LFP values were used to align events. Sensory and motor events were then used as triggers to compute mean LFP, CSD maps and PETHs of MUA. Onsets and offsets of sensory and short-lasting motor events were estimated using the trapezoidal sum-based method on triggered, spike histograms. Mega-bursts onsets and offsets were simply estimated by the first and last local minimum LFP value.

Cross-correlation analysis of spike times, and bursts times (onset phase) in the two different zones was carried out essentially as described previously.

### Statistics

We did not use statistical methods to calculate the required sample size, *a priori*, but the number of animals used in this study is consistent with that used in previous reports^13^. Data could not be acquired by an experimenter blinded to the study design. Nonetheless, data were analysed offline, in serial runs, and was independently verified by different analysts. Unless otherwise indicated, the statistical tests used do not assume a normal distribution of the data, and to compare two groups, we used the Wilcoxon rank-sum test (unpaired data) or the Wilcoxon signed-rank test (paired-data), as indicated in the ‘Results’ section and figure captions. In some cases (twitches-related within-bursts MUA peak frequency and respective time in relation to twitch onset, as well as bursts offset, that is, peak frequency to baseline return, twitch/complex movements, maximal amplitudes, power and durations, before and after deafferentation), the Kolmogorov–Smirnov test was used instead. In additional particular cases, namely comparisons relative to firing, bursting or movement rates before and after deafferentation, to test for significance, we used a shuffling/bootstrap method. For each animal, we randomly selected 30 recorded minutes. The spikes/bursts train in a selected sensory or motor channel was circularized, and the beginning of a trial was randomly chosen. Then, surrogate trials were recomputed 100 times to generate a global band of confidence (the 5% highest and lowest values of the 100 trials × 3 animals). Results were considered significant when *P*<0.05; in the figures, *P*<0.05, *P*<0.01 and *P*<0.001 were represented by one, two or three asterisks, respectively; specific *P* were included in the ‘Results’ section.

### Data availability

The authors declare that all data and MATLAB codes used to generate the figures are available upon request.

## Additional information

**How to cite this article:** Inácio, A. R. *et al*. Sensory feedback synchronizes motor and sensory neuronal networks in the neonatal rat spinal cord. *Nat. Commun.* **7,** 13060 doi: 10.1038/ncomms13060 (2016).

## Supplementary Material

Supplementary Information Supplementary Figures 1 - 7 and Supplementary References

## References

[b1] Blumberg M. S., Freeman J. H. & Robinson S. R. Oxford Handbook of Developmental Behavioral Neuroscience Oxford University Press (2010).

[b2] Hamburger V. in The Mammalian Fetus: Comparative Biology and Methodology ed. Hafez E. S. 69–81Charles C. Thomas (1975).

[b3] De Vries J. I., Visser G. H. & Prechtl H. F. The emergence of fetal behaviour. I. Qualitative aspects. Early Hum. Dev. 7, 301–322 (1982). 7169027 10.1016/0378-3782(82)90033-0

[b4] Robinson S. R., Blumberg M. S., Lane M. S. & Kreber L. A. Spontaneous motor activity in fetal and infant rats is organized into discrete multilimb bouts. Behav. Neurosci. 114, 328–336 (2000). 10832794 10.1037//0735-7044.114.2.328

[b5] Kreider J. C. & Blumberg M. S. Mesopontine contribution to the expression of active ‘twitch’ sleep in decerebrate week-old rats. Brain Res. 872, 149–159 (2000). 10924687 10.1016/s0006-8993(00)02518-x

[b6] Del Rio-Bermudez C., Sokoloff G. & Blumberg M. S. Sensorimotor processing in the newborn rat red nucleus during active sleep. J. Neurosci. 35, 8322–8332 (2015). 26019345 10.1523/JNEUROSCI.0564-15.2015 PMC4444549

[b7] Waldenstrom A., Christensson M. & Schouenborg J. Spontaneous movements: Effect of denervation and relation to the adaptation of nociceptive withdrawal reflexes in the rat. Physiol. Behav. 98, 532–536 (2009). 19715712 10.1016/j.physbeh.2009.08.009

[b8] Sanes J. R. & Lichtman J. W. Development of the vertebrate neuromuscular junction. Annu. Rev. Neurosci. 22, 389–442 (1999). 10202544 10.1146/annurev.neuro.22.1.389

[b9] Petersson P., Waldenstrom A., Fahraeus C. & Schouenborg J. Spontaneous muscle twitches during sleep guide spinal self-organization. Nature 424, 72–75 (2003). 12840761 10.1038/nature01719

[b10] Mendelsohn A. I., Simon C. M., Abbott L. F., Mentis G. Z. & Jessell T. M. Activity regulates the incidence of heteronymous sensory-motor connections. Neuron. 87, 111–123 (2015). 26094608 10.1016/j.neuron.2015.05.045 PMC4504246

[b11] Yang J. W., Hanganu-Opatz I. L., Sun J. J. & Luhmann H. J. Three patterns of oscillatory activity differentially synchronize developing neocortical networks in vivo. J. Neurosci. 29, 9011–9025 (2009). 19605639 10.1523/JNEUROSCI.5646-08.2009 PMC6665441

[b12] Marcano-Reik A. J. & Blumberg M. S. The corpus callosum modulates spindle-burst activity within homotopic regions of somatosensory cortex in newborn rats. Eur. J. Neurosci. 28, 1457–1466 (2008). 18973571 10.1111/j.1460-9568.2008.06461.x PMC2669778

[b13] Khazipov R. . Early motor activity drives spindle bursts in the developing somatosensory cortex. Nature 432, 758–761 (2004). 15592414 10.1038/nature03132

[b14] Yang J. W. . Thalamic network oscillations synchronize ontogenetic columns in the newborn rat barrel cortex. Cereb. Cortex. 23, 1299–1316 (2013). 22593243 10.1093/cercor/bhs103

[b15] Milh M. . Rapid cortical oscillations and early motor activity in premature human neonate. Cereb. Cortex. 17, 1582–1594 (2007). 16950867 10.1093/cercor/bhl069

[b16] Vanhatalo S. & Kaila K. Development of neonatal EEG activity: from phenomenology to physiology. Semin. Fetal Neonatal Med. 11, 471–478 (2006). 17018268 10.1016/j.siny.2006.07.008

[b17] An S. M., Kilb W. & Luhmann H. J. Sensory-evoked and spontaneous gamma and spindle bursts in neonatal rat motor cortex. J. Neurosci. 34, 10870–10883 (2014). 25122889 10.1523/JNEUROSCI.4539-13.2014 PMC6705262

[b18] McVea D. A., Mohajerani M. H. & Murphy T. H. Voltage-sensitive dye imaging reveals dynamic spatiotemporal properties of cortical activity after spontaneous muscle twitches in the newborn rat. J. Neurosci. 32, 10982–10994 (2012). 22875932 10.1523/JNEUROSCI.1322-12.2012 PMC6621025

[b19] Blankenship A. G. & Feller M. B. Mechanisms underlying spontaneous patterned activity in developing neural circuits. Nat. Rev. Neurosci. 11, 18–29 (2010). 19953103 10.1038/nrn2759 PMC2902252

[b20] Moody W. J. & Bosma M. M. Ion channel development, spontaneous activity, and activity-dependent development in nerve and muscle cells. Physiol. Rev. 85, 883–941 (2005). 15987798 10.1152/physrev.00017.2004

[b21] O’Donovan M. J., Chub N. & Wenner P. Mechanisms of spontaneous activity in developing spinal networks. J. Neurobiol. 37, 131–145 (1998). 9777737 10.1002/(sici)1097-4695(199810)37:1<131::aid-neu10>3.0.co;2-h

[b22] Marder E. & Rehm K. J. Development of central pattern generating circuits. Curr. Opin. Neurobiol. 15, 86–93 (2005). 15721749 10.1016/j.conb.2005.01.011

[b23] Clancy B., Darlington R. B. & Finlay B. L. Translating developmental time across mammalian species. Neuroscience 105, 7–17 (2001). 11483296 10.1016/s0306-4522(01)00171-3

[b24] Marques H. G., Bharadwaj A. & Iida F. From spontaneous motor activity to coordinated behaviour: a developmental model. PLoS Comput. Biol. 10, e1003653 (2014). 25057775 10.1371/journal.pcbi.1003653 PMC4109855

[b25] Blumberg M. S. . Development of twitching in sleeping infant mice depends on sensory experience. Curr. Biol. 25, 656–662 (2015). 25702578 10.1016/j.cub.2015.01.022 PMC4348337

[b26] Waldenstrom A., Thelin J., Thimansson E., Levinsson A. & Schouenborg J. Developmental learning in a pain-related system: evidence for a cross-modality mechanism. J. Neurosci. 23, 7719–7725 (2003). 12930812 10.1523/JNEUROSCI.23-20-07719.2003 PMC6740755

[b27] Schouenborg J. & Weng H. R. Sensorimotor transformation in a spinal motor system. Exp. Brain. Res. 100, 170–174 (1994). 7813646 10.1007/BF00227291

[b28] Minlebaev M., Colonnese M., Tsintsadze T., Sirota A. & Khazipov R. Early gamma oscillations synchronize developing thalamus and cortex. Science 334, 226–229 (2011). 21998388 10.1126/science.1210574

[b29] Mohns E. J. & Blumberg M. S. Synchronous bursts of neuronal activity in the developing hippocampus: modulation by active sleep and association with emerging gamma and theta rhythms. J. Neurosci. 28, 10134–10144 (2008). 18829971 10.1523/JNEUROSCI.1967-08.2008 PMC2678192

[b30] Tiriac A., Del Rio-Bermudez C. & Blumberg M. S. Self-generated movements with “unexpected” sensory consequences. Curr. Biol. 24, 2136–2141 (2014). 25131675 10.1016/j.cub.2014.07.053 PMC4175005

[b31] Crapse T. B. & Sommer M. A. Corollary discharge across the animal kingdom. Nat. Rev. Neurosci. 9, 587–600 (2008). 18641666 10.1038/nrn2457 PMC5153363

[b32] Rudomin P. & Schmidt R. F. Presynaptic inhibition in the vertebrate spinal cord revisited. Exp. Brain. Res. 129, 1–37 (1999). 10550500 10.1007/s002210050933

[b33] Fellippa-Marques S., Vinay L. & Clarac F. Spontaneous and locomotor-related GABAergic input onto primary afferents in the neonatal rat. Eur. J. Neurosci. 12, 155–164 (2000). 10651870 10.1046/j.1460-9568.2000.00895.x

[b34] Bos R., Brocard F. & Vinay L. Primary afferent terminals acting as excitatory interneurons contribute to spontaneous motor activities in the immature spinal cord. J. Neurosci. 31, 10184–10188 (2011). 21752994 10.1523/JNEUROSCI.0068-11.2011 PMC6623060

[b35] Kremer E. & Lev-Tov A. GABA-receptor-independent dorsal root afferents depolarization in the neonatal rat spinal cord. J. Neurophysiol. 79, 2581–2592 (1998). 9582230 10.1152/jn.1998.79.5.2581

[b36] Fitzgerald M. The post-natal development of cutaneous afferent fibre input and receptive field organization in the rat dorsal horn. J. Physiol. 364, 1–18 (1985). 4032293 10.1113/jphysiol.1985.sp015725 PMC1192950

[b37] Fitzgerald M. & Jennings E. The postnatal development of spinal sensory processing. Proc. Natl. Acad. Sci. USA 96, 7719–7722 (1999). 10393887 10.1073/pnas.96.14.7719 PMC33608

[b38] Fitzgerald M. The development of nociceptive circuits. Nat. Rev. Neurosci. 6, 507–520 (2005). 15995722 10.1038/nrn1701

[b39] Friede R. L. & Samorajski T. Myelin formation in the sciatic nerve of the rat. A quantitative electron microscopic, histochemical and radioautographic study. J. Neuropathol. Exp. Neurol. 27, 546–570 (1968). 4879906

[b40] Webster H. D. The geometry of peripheral myelin sheaths during their formation and growth in rat sciatic nerves. J. Cell Biol. 48, 348–367 (1971). 4928020 10.1083/jcb.48.2.348 PMC2108190

[b41] Wills T. J., Muessig L. & Cacucci F. The development of spatial behaviour and the hippocampal neural representation of space. Philos. Trans. R Soc. Lond. B Biol. Sci. 369, 20130409 (2014). 24366148 10.1098/rstb.2013.0409 PMC3866458

[b42] Blumberg M. S. Beyond dreams: do sleep-related movements contribute to brain development? Front Neurol. 1, 140 (2010). 21344014 10.3389/fneur.2010.00140 PMC3034236

[b43] Demir R., Gao B. X., Jackson M. B. & Ziskind-Conhaim L. Interactions between multiple rhythm generators produce complex patterns of oscillation in the developing rat spinal cord. J. Neurophysiol. 87, 1094–1105 (2002). 11826073 10.1152/jn.00276.2001

[b44] Bracci E., Ballerini L. & Nistri A. Localization of rhythmogenic networks responsible for spontaneous bursts induced by strychnine and bicuculline in the rat isolated spinal cord. J. Neurosci. 16, 7063–7076 (1996). 8824342 10.1523/JNEUROSCI.16-21-07063.1996 PMC6579249

[b45] Tiriac A., Uitermarkt B. D., Fanning A. S., Sokoloff G. & Blumberg M. S. Rapid whisker movements in sleeping newborn rats. Curr. Biol. 22, 2075–2080 (2012). 23084988 10.1016/j.cub.2012.09.009 PMC3494768

[b46] Fulton B. P. Postnatal changes in conduction velocity and soma action potential parameters of rat dorsal root ganglion neurones. Neurosci. Lett. 73, 125–130 (1987). 3822244 10.1016/0304-3940(87)90005-x

[b47] Sima A. Studies on fibre size in developing sciatic nerve and spinal roots in normal, undernourished, and rehabilitated rats. Acta Physiol. Scand Suppl. 406, 1–55 (1974). 4526498

[b48] Colonnese M. T. . A conserved switch in sensory processing prepares developing neocortex for vision. Neuron. 67, 480–498 (2010). 20696384 10.1016/j.neuron.2010.07.015 PMC2946625

[b49] Ben-Ari Y. Developing networks play similar melody. Trends. Neurosci. 24, 353–360 (2001). 11356508 10.1016/s0166-2236(00)01813-0

[b50] Minlebaev M., Ben Ari Y. & Khazipov R. NMDA receptors pattern early activity in the developing barrel cortex in vivo. Cereb. Cortex. 19, 688–696 (2009). 18663251 10.1093/cercor/bhn115

[b51] Ren J. & Greer J. J. Ontogeny of rhythmic motor patterns generated in the embryonic rat spinal cord. J. Neurophysiol. 89, 1187–1195 (2003). 12626606 10.1152/jn.00539.2002

[b52] Myers C. P. . Cholinergic input is required during embryonic development to mediate proper assembly of spinal locomotor circuits. Neuron. 46, 37–49 (2005). 15820692 10.1016/j.neuron.2005.02.022

[b53] Duchen M. R. Excitation of mouse motoneurones by GABA-mediated primary afferent depolarization. Brain Res. 379, 182–187 (1986). 3017508 10.1016/0006-8993(86)90274-x

[b54] Gao B. X. & Ziskind-Conhaim L. Development of glycine-and GABA-gated currents in rat spinal motoneurons. J. Neurophysiol. 74, 113–121 (1995). 7472315 10.1152/jn.1995.74.1.113

[b55] Wu W. L., Ziskind-Conhaim L. & Sweet M. A. Early development of glycine- and GABA-mediated synapses in rat spinal cord. J. Neurosci. 12, 3935–3945 (1992). 1403091 10.1523/JNEUROSCI.12-10-03935.1992 PMC6575960

[b56] Jean-Xavier C., Pflieger J. F., Liabeuf S. & Vinay L. Inhibitory postsynaptic potentials in lumbar motoneurons remain depolarizing after neonatal spinal cord transection in the rat. J. Neurophysiol. 96, 2274–2281 (2006). 16807348 10.1152/jn.00328.2006

[b57] Baccei M. L. & Fitzgerald M. Development of GABAergic and glycinergic transmission in the neonatal rat dorsal horn. J. Neurosci. 24, 4749–4757 (2004). 15152035 10.1523/JNEUROSCI.5211-03.2004 PMC6729459

[b58] Stil A. . Developmental up-regulation of the potassium-chloride cotransporter type 2 in the rat lumbar spinal cord. Neuroscience. 164, 809–821 (2009). 19699273 10.1016/j.neuroscience.2009.08.035

[b59] Minlebaev M., Ben-Ari Y. & Khazipov R. Network mechanisms of spindle-burst oscillations in the neonatal rat barrel cortex in vivo. J. Neurophysiol. 97, 692–700 (2007). 17093125 10.1152/jn.00759.2006

[b60] Valeeva G., Tressard T., Mukhtarov M., Baude A. & Khazipov R. An optogenetic approach for investigation of excitatory and inhibitory network GABA actions in mice expressing channelrhodopsin-2 in GABAergic neurons. J. Neurosci. 36, 5961–5973 (2016). 27251618 10.1523/JNEUROSCI.3482-15.2016 PMC6601813

[b61] Kirmse K. . GABA depolarizes immature neurons and inhibits network activity in the neonatal neocortex in vivo. Nat. Commun. 6, 7750 (2015). 26177896 10.1038/ncomms8750

[b62] Cunningham M. G., Donalds R. A., Scouten C. W. & Tresch M. C. A versatile, low-cost adaptor for stereotaxic and electrophysiologic spinal preparations in juvenile and adult rodents. Brain Res. Bull. 68, 157–162 (2005). 16325015 10.1016/j.brainresbull.2005.08.004

[b63] Farrar M. J. . Chronic in vivo imaging in the mouse spinal cord using an implanted chamber. Nat. Methods. 9, 297–302 (2012). 22266542 10.1038/nmeth.1856 PMC3429123

[b64] Fenrich K. K. . Long-term in vivo imaging of normal and pathological mouse spinal cord with subcellular resolution using implanted glass windows. J. Physiol. 590, 3665–3675 (2012). 22641787 10.1113/jphysiol.2012.230532 PMC3476626

[b65] Graham B. A., Brichta A. M. & Callister R. J. An in vivo mouse spinal cord preparation for patch-clamp analysis of nociceptive processing. J. Neurosci. Methods. 136, 221–228 (2004). 15183274 10.1016/j.jneumeth.2004.01.014

[b66] Karlsson K. A. & Blumberg M. S. The union of the state: myoclonic twitching is coupled with nuchal muscle atonia in infant rats. Behav. Neurosci. 116, 912–917 (2002). 12369810 10.1037//0735-7044.116.5.912

[b67] Tsintsadze V., Minlebaev M., Suchkov D., Cunningham M. O. & Khazipov R. Ontogeny of kainate-induced gamma oscillations in the rat CA3 hippocampus in vitro. Front Cell Neurosci. 9, 195 (2015). 26041996 10.3389/fncel.2015.00195 PMC4438719

[b68] Mitrukhina O., Suchkov D., Khazipov R. & Minlebaev M. Imprecise whisker map in the neonatal rat barrel cortex. Cereb. Cortex. 25, 3458–3467 (2015). 25100857 10.1093/cercor/bhu169

[b69] Blumberg M. S., Coleman C. M., Gerth A. I. & McMurray B. Spatiotemporal structure of REM sleep twitching reveals developmental origins of motor synergies. Curr. Biol. 23, 2100–2109 (2013). 24139739 10.1016/j.cub.2013.08.055 PMC3823644

[b70] Kaneoke Y. & Vitek J. L. Burst and oscillation as disparate neuronal properties. J. Neurosci Methods 68, 211–223 (1996). 8912194 10.1016/0165-0270(96)00081-7

