# Supplementary material for: Sensory feedback synchronizes motor and sensory neuronal networks in the neonatal rat spinal cord
Source: Nat Commun. 2016 Oct 7;7:13060. doi: 10.1038/ncomms13060 (PMC5494195; doi:10.1038/ncomms13060)
Supplement: Supplementary Information — Supplementary Figures 1 - 7 and Supplementary References [file ncomms13060-s1.pdf]

Supplementary figures and figure legends

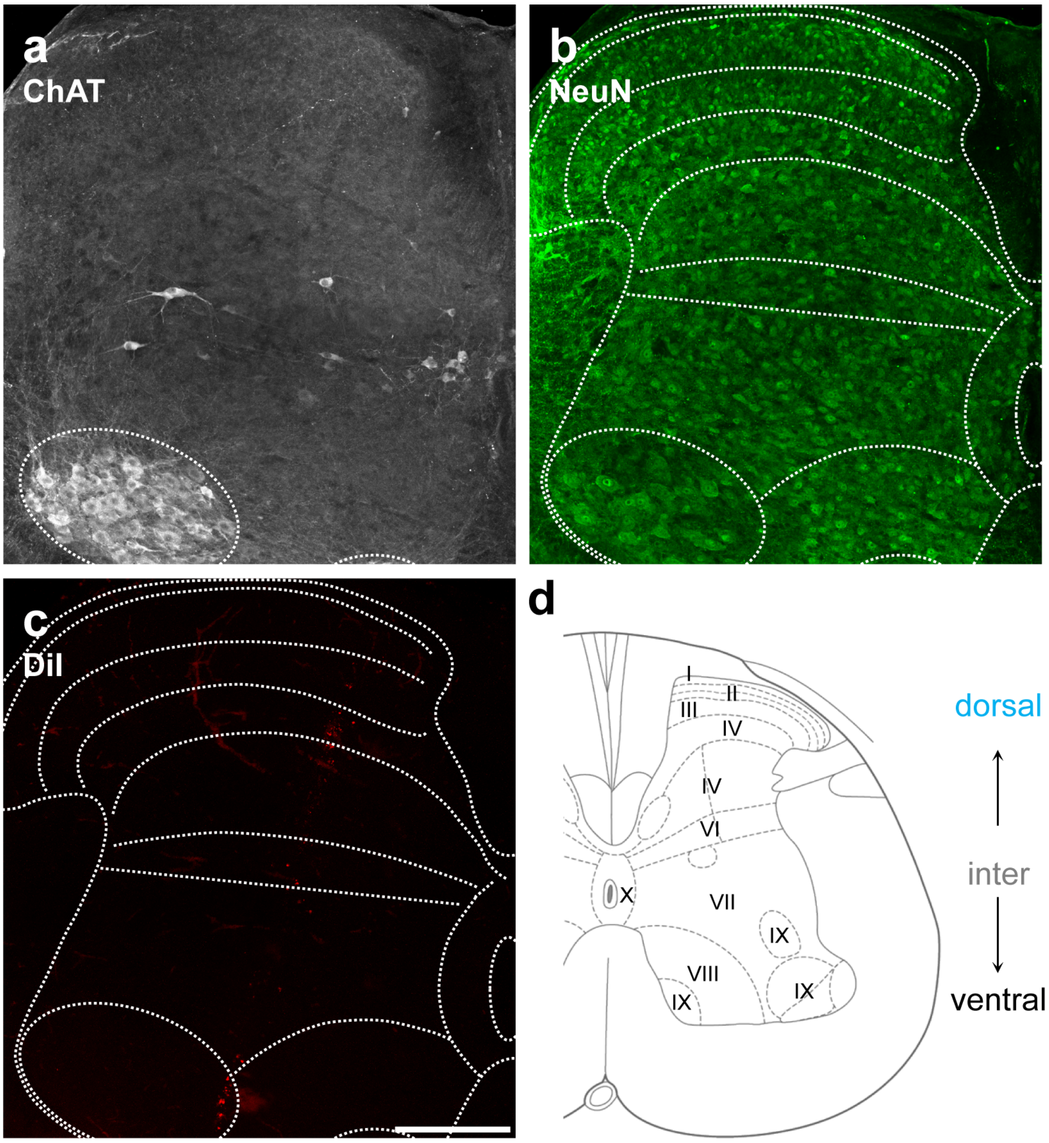

Supplementary Figure 1| Histological analysis. (a-c) Confocal micrographs (Z-projections) of ChAT (Alexa633, gray-scale), NeuN (Alexa488, green) and DiI (red) signals within a transverse spinal cord slice (same as in Fig. 1b, L3). Scale bar: 200 µm. **(d)** Anatomical organization of the lumbar spinal cord (segment L3) in adult rats (adapted from[1]). These data were used to extrapolate the location of each recording site, particularly with respect to different spinal cord laminae.

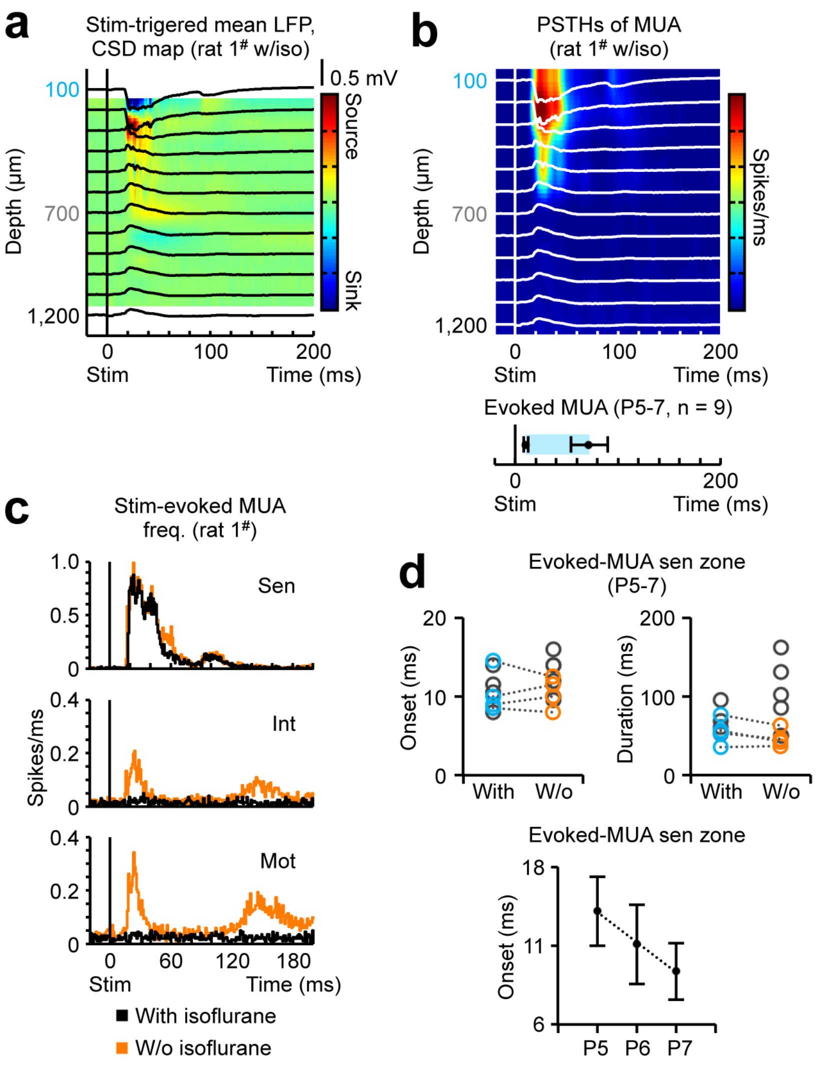

Supplementary Figure 2| Isoflurane suppresses ventral horn activation by sensory stimulation. (a) Mechanical stimulus (stim)-triggered mean LFP traces and CSD map (n ≈ 100 stimuli, single anesthetized animal - 0.5 % isoflurane). Changes in extracellular potential are qualitatively equivalent to those observed when the animals were not administered isoflurane (Fig. 2b, same animal and stimulation point). **(b)** Corresponding normalized PSTHs of MUA. **c)** Normalized PSTHs of MUA in sensory zone (sen, depths of 100 and 200 µm), intermediate zone (int, depths of 800 and 900 µm) and motor zone (mot, depths of 1100 and 1200 µm) in the presence and absence of isoflurane (black and orange, respectively, single animal). As evidenced in **a-c**, isoflurane suppresses stim-triggered ventral horn activation. **d)** Top: Stim-evoked sen MUA burst onset and duration under 0.5 % or no isoflurane per animal (n = 9-10 for each of the conditions tested); in 4 animals, responses

were recorded in both conditions (blue and orange data points, respectively). No difference was found when comparing within-burst MUA frequency (paired data, n = 4, paired two-tailed Student's *t*-test), bursts onset and duration (pooled data, n = 10 with isoflurane and n = 9 without isoflurane, Wilcoxon rank-sum test). These results are consistent with a previous study showing that isoflurane at low concentrations (< 1.5 %) does not produce a significant effect over cortical sensory responses in neonatal rats (first postnatal week)[2]. Bottom: Stim-triggered MUA burst onsets per age group in pooled isoflurane and non-isoflurane data (mean ± s.d., 4-6 animals per age group, P = 0.0531, Kruskal-Wallis test).

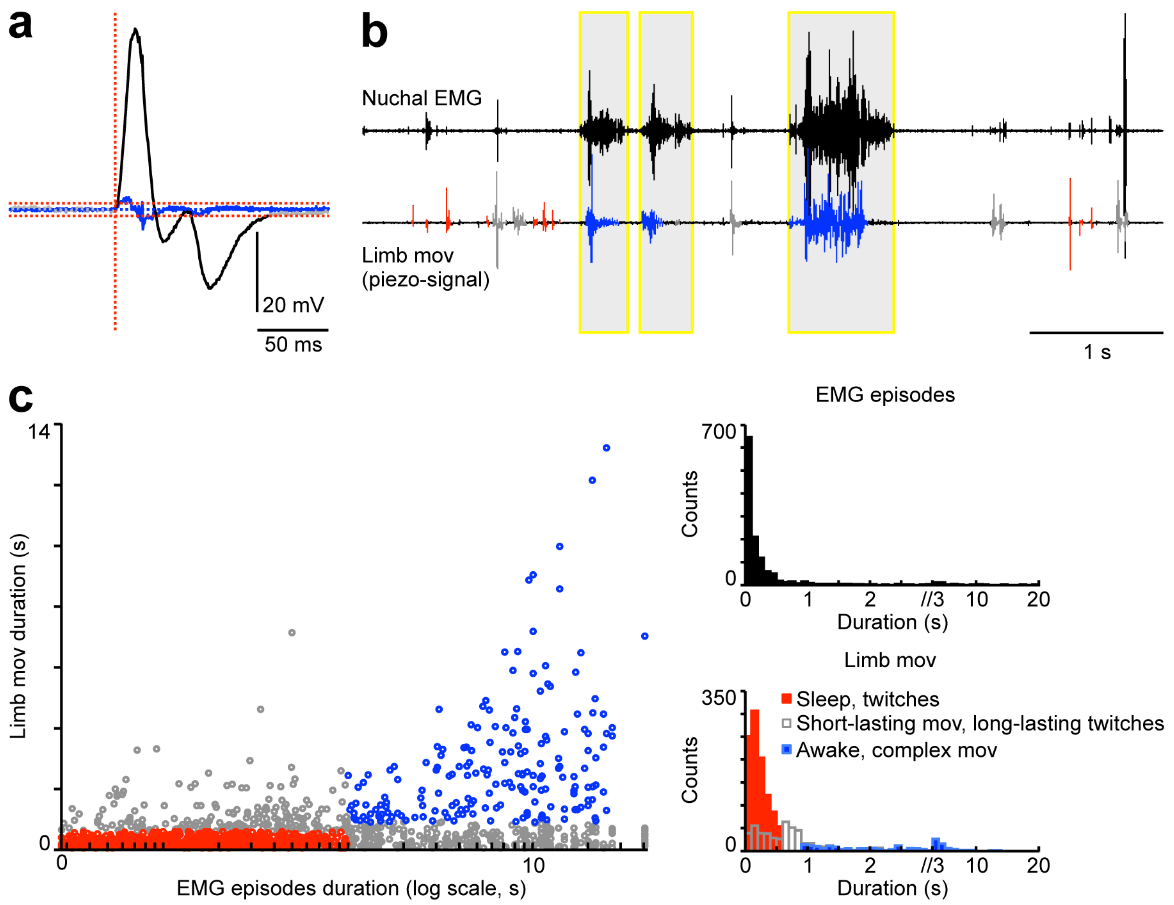

Supplementary Figure 3| Movement and sleep-awake cycles detection. (a) Exemplification of the semi-automatic movement detection; original limb movement signal (gray: baseline, black: detected twitch; red horizontal dashed lines: threshold; blue horizontal line: signal first-derivative; red vertical dashed line: onset). All detected events lasting less than 600 ms were considered putative twitches. Putative twitches were further classified, manually, as twitches or short-lasting movements based on existence of preceding or following activity. Events lasting more than 900 ms were considered complex movements. **(b)** Traces exemplifying simultaneously recorded nuchal EMG (gray bars denote EMG events lasting more than 1 s, considered as awake states) and piezo-derived limb movements (mov) signal (red: events lasting less than 600 ms, associated to EMG events lasting up to 1 s; blue: events lasting more than 900 ms, associated to EMG events lasting more than 1 s; gray: all others). **(c)** Scatter plot of durations of detected episodes of nuchal EMG activity associated with

overt limb movement(s), and respective histograms (n = 1237 EMG activity bouts and 1698 movements of 3 animals).

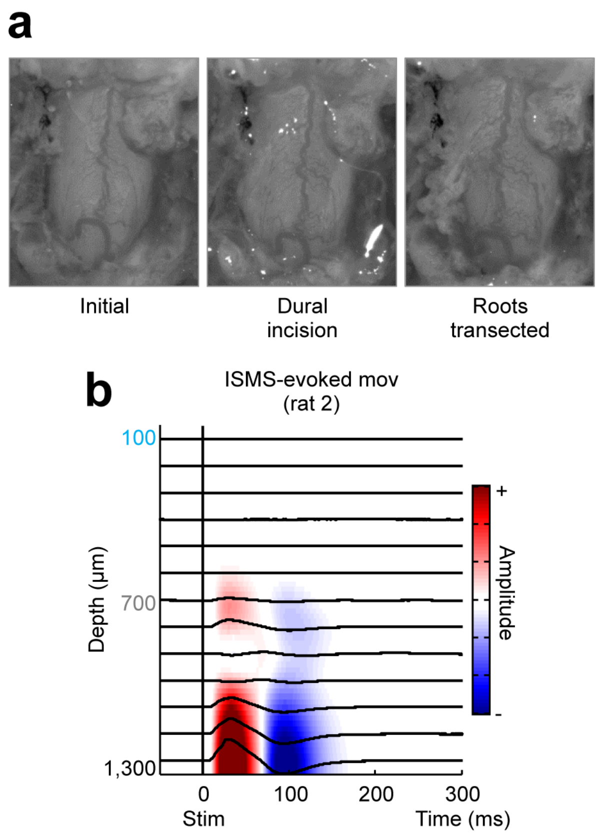

Supplementary Figure 4| *In vivo* local deafferentation procedure. a) Macroscopic pictures representing the local deafferentation performed, in this case, in a P7 animal. We first made a rostral-caudal dura matter incision, over the exposed left-most portion of the tissue; the otherwise tight structural organization of the soft tissues was slightly relieved by incision, and afferents tended to fall aside from the spinal cord, allowing us to delicately approach, using a ultra-fine micro forceps, and very gently clipping, using an ultra-fine micro scissors, those fibers. Left: View of the dorsal spinal cord; intact dura matter. Middle: Dural incision, followed by natural protrusion of dorsal rootlets. Right: Transected rootlets. **(b)** Mean movement (mov) traces obtained for each depth of ISMS ($n \approx 50$ pulses per depth, under 1.5 % isoflurane) overlaid on a respective normalized (color-coded) amplitude map (same animal as in Fig. 3b, d-e). ISMS was performed upon conclusion of post-deafferentation recordings.

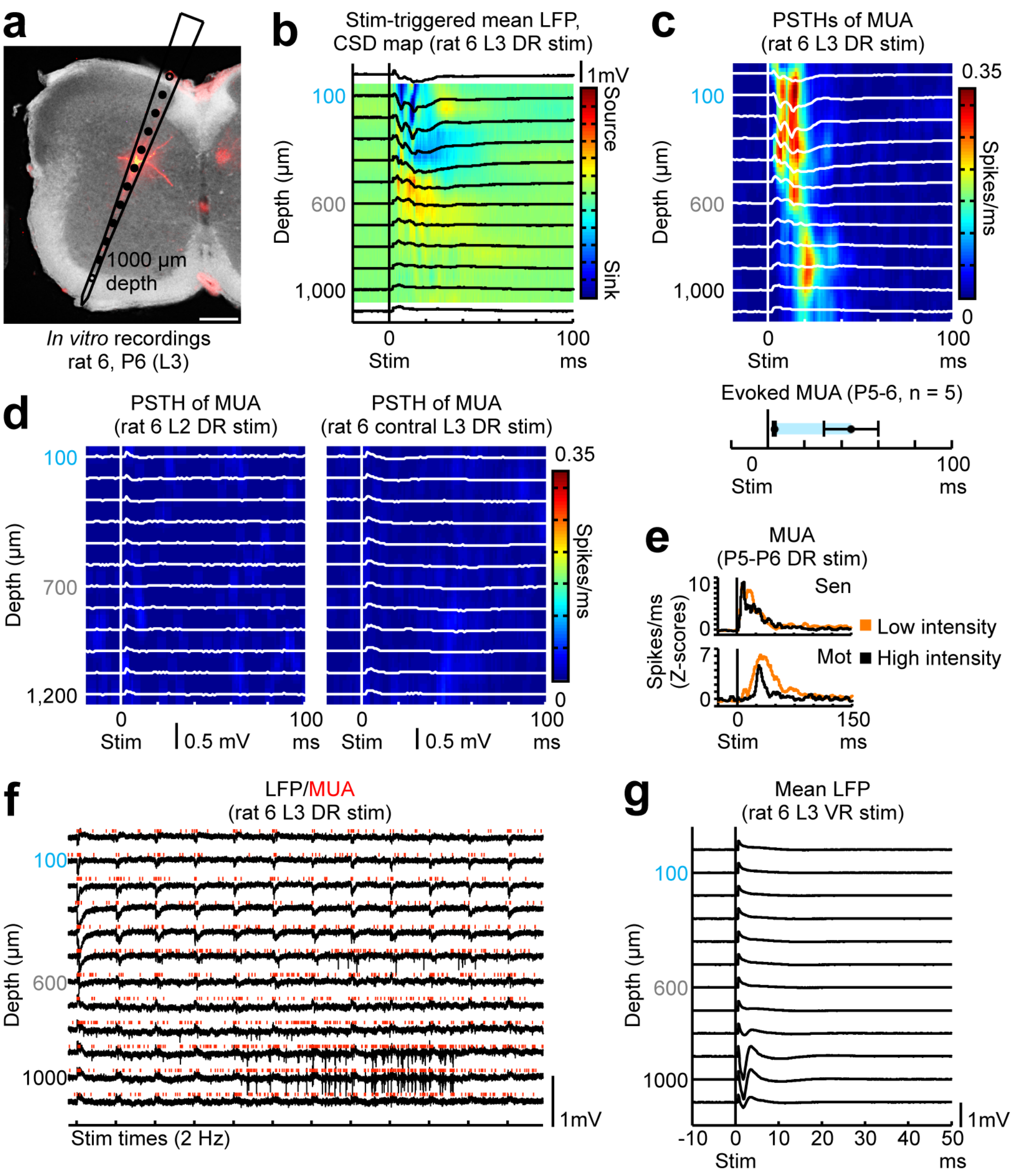

Supplementary Figure 5| *In vitro* activity patterns induced by dorsal root stimulation. (a) Silicone-based electrode array track visualized on a transverse spinal cord section (P6 rat). Overlaid dark-field image and epifluorescence image (DiI, red) and to scale scheme of the electrode array, representing the typical anatomical positioning of different recordings sites in the *in vitro* spinal cord preparation (in this case, L3 segment), equivalent to that of the *in vivo* experiments. **(b)** Dorsal root (L3) stimulation (stim)-triggered mean LFP and CSD map (n ≈ 50 stimuli, single animal). Note the short-latency LFP deflections and current sinks evoked in the dorsal horn (sensory zone). **c)** Associated normalized PSTHs of MUA, and average MUA response times, with graphed onsets and offsets

(mean ± s.d., n = 5 animals). The sensory potential was associated with a MUA burst, at a latency of 3.6 ± 0.65 ms and lasting 42 ± 14 ms post-stimulus (mean ± s.d., n = 5 animals), essentially limited to the dorsal horn (depth range of 100 - 400 µm). **d)** Anterior dorsal root (L2) and contralateral dorsal root (L3) stim-triggered mean LFP and PSTH of MUA (n ≈ 50 stimuli, same animal as in **c**). Note that equivalent stimulations of anterior, posterior or contralateral roots evoked no or a weaker spinal response. **e)** Normalized PSTHs of MUA (Z-scores) demonstrating the effect of stim on firing rates in sensory (sen) and motor (mot) zones (mean of 3 animals). **f)** Original wide-band LFP traces denoting spinal responses to rhythmic dorsal root stimulation (single animal). Mot zones were weakly activated by single stimulus. However, rhythmic stimulation of dorsal roots (at least 5 stimuli at 1-2 Hz) evoked long-lasting rhythmic oscillatory bursts in mot zones, with coherence between mot units and oscillation. **g)** Ventral root (L3) stim-triggered mean LFP traces (n = 50 stimuli, single animal), revealing clear anterograde population spikes in ventral horn (mot zone). Generally, electrical stimulation of the respective ventral root evoked anterograde population spikes in the ventral horn (depth range: 900 - 1100 µm, n = 5 animals), showing peak amplitude at 2.8 ± 0.45 ms after stimulus (mean ± s.d., n = 5 animals).

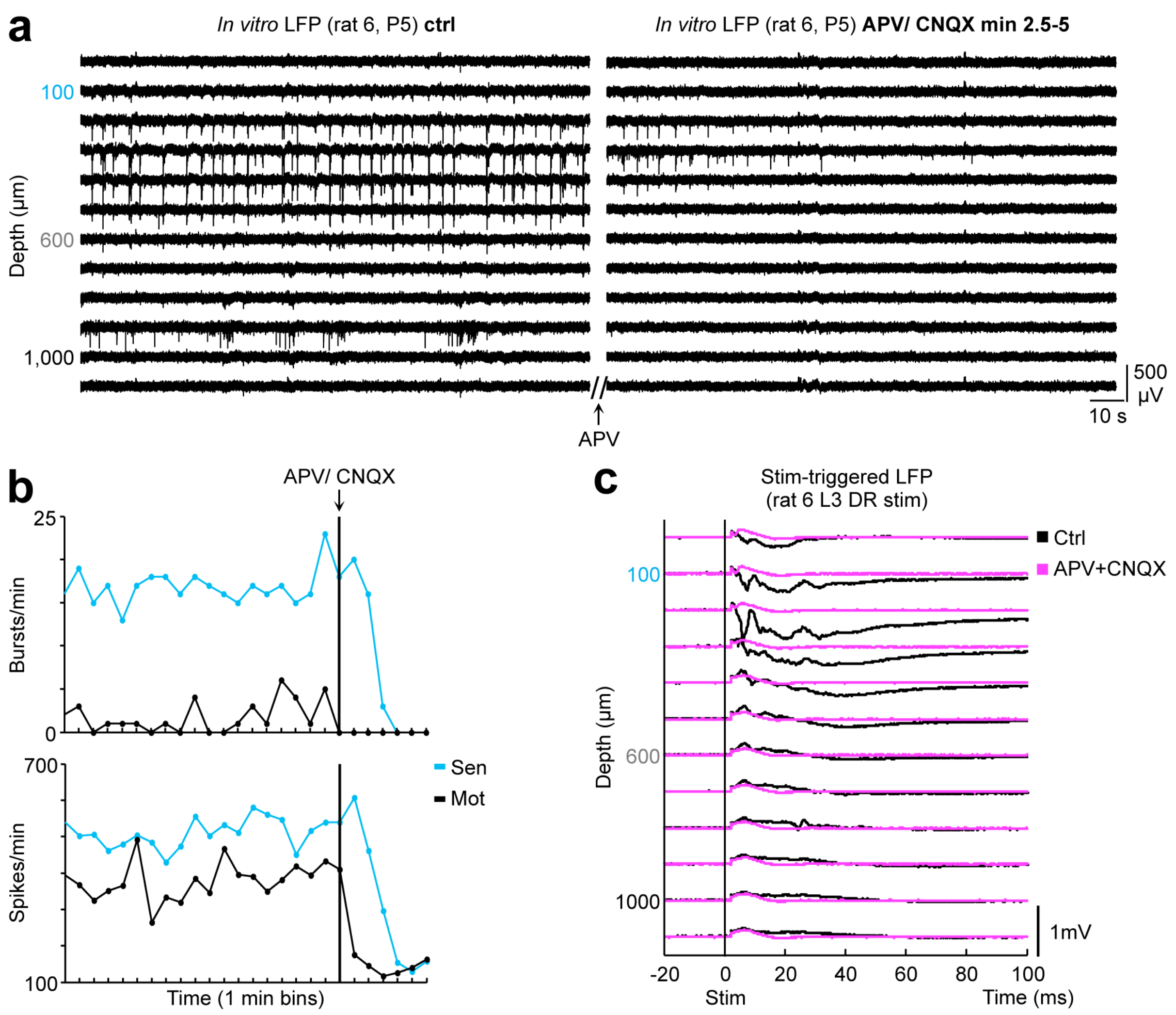

Supplementary Figure 6| Blockade of sensory and motor network events, *in vitro*, by APV and CNQX. a) Original wide-band LFP traces exemplifying translaminar spontaneous spinal cord activity dynamics, *in vitro*, before and following bath application of the ionotropic glutamate receptor antagonists APV and CNQX, with suppression of network events in the after condition (single animal). **b)** Respective time course of APV and CNQX effect on bursting and spiking activity within the sensory zone (sen, light blue) and motor zone (mot, black). **c)** Dorsal root (L3) stimulation (stim)-triggered mean LFP traces across different laminae before (black) and after (magenta) APV and CNQX application (n ≈ 50 stimuli, single animal). Response suppression in the after condition confirmed its dependence on synaptic transmission.

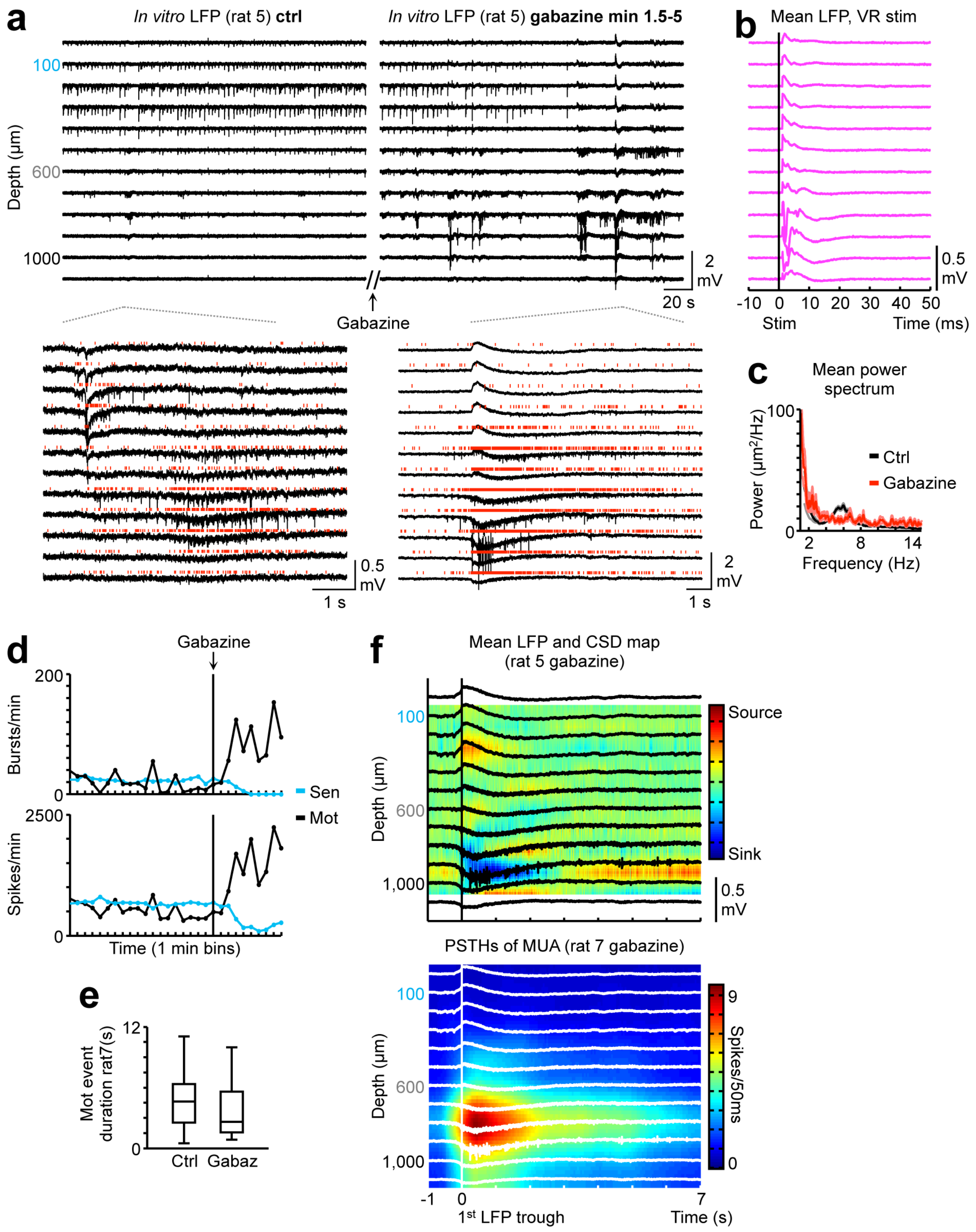

Supplementary Figure 7| *In vitro* spontaneous patterns of activity: suppression of sensory events and emergence of epileptiform motor events with gabazine. (a) Top: Original wide-band LFP traces across different spinal cord laminae before and following bath application of the GABA(A) receptor

antagonist gabazine (single animal), demonstrating the observed drug-induced suppression of sensory events and emergence of epileptiform motor events. Bottom: Exemplary motor events prior to and after gabazine application. **(b)** Ventral root stimulation (stim)-triggered mean LFP traces, identifying motor neuron pool depths (n ≈ 50 stimuli, single animal). **(c)** Mean power spectrum of detected bursts referent to a selected motor zone (mot) recording site before and after gabazine application (mean ± s.d., single animal), showing a loss of event structural stereotypy in the presence of the GABA(A) receptor antagonist. **(d)** Respective time course of gabazine effect on bursting and spiking activity within the sensory zone (sen, light blue) and mot (black). **(e)** Motor event duration for the before and after conditions (mean ± s.d., single animal). **(f)** Top: Mean LFP triggered by motor burst occurring in the presence of gabazine and CSD map. Bottom: Corresponding normalized PETHs of MUA (single animal).

Supplementary references

1. Watson, C., Paxinos, G. & Kayalioglu, G. *The Spinal Cord, 1st Edition: a Christopher and Dana Reeve Foundation Text and Atlas.* 290 (Academic Press, 2009).

2. Sitdikova, G. et al. Isoflurane suppresses early cortical activity. *Ann. Clin. Transl. Neurol.* **1,** 15-26 (2014).
